# Comprehensive analysis of epigenetic clocks reveals associations between disproportionate biological ageing and hippocampal volume

**DOI:** 10.1007/s11357-022-00558-8

**Published:** 2022-04-21

**Authors:** Lidija Milicic, Michael Vacher, Tenielle Porter, Vincent Doré, Samantha C. Burnham, Pierrick Bourgeat, Rosita Shishegar, James Doecke, Nicola J. Armstrong, Rick Tankard, Paul Maruff, Colin L. Masters, Christopher C. Rowe, Victor L. Villemagne, Simon M. Laws, Alzheimer’s Disease Neuroimaging Initiative (ADNI), Michael Weiner, Paul Aisen, Ronald Petersen, Clifford R. Jack, William Jagust, John Q. Trojanowki, Arthur W. Toga, Laurel Beckett, Robert C. Green, Andrew J. Saykin, John C. Morris, Leslie M. Shaw, Enchi Liu, Tom Montine, Ronald G. Thomas, Michael Donohue, Sarah Walter, Devon Gessert, Tamie Sather, Gus Jiminez, Danielle Harvey, Matthew Bernstein, Nick Fox, Paul Thompson, Norbert Schuff, Charles DeCArli, Bret Borowski, Jeff Gunter, Matt Senjem, Prashanthi Vemuri, David Jones, Kejal Kantarci, Chad Ward, Robert A. Koeppe, Norm Foster, Eric M. Reiman, Kewei Chen, Chet Mathis, Susan Landau, Nigel J. Cairns, Erin Householder, Lisa Taylor Reinwald, Virginia Lee, Magdalena Korecka, Michal Figurski, Karen Crawford, Scott Neu, Tatiana M. Foroud, Steven Potkin, Li Shen, Faber Kelley, Sungeun Kim, Kwangsik Nho, Zaven Kachaturian, Richard Frank, Peter J. Snyder, Susan Molchan, Jeffrey Kaye, Joseph Quinn, Betty Lind, Raina Carter, Sara Dolen, Lon S. Schneider, Sonia Pawluczyk, Mauricio Beccera, Liberty Teodoro, Bryan M. Spann, James Brewer, Helen Vanderswag, Adam Fleisher, Judith L. Heidebrink, Joanne L. Lord, Ronald Petersen, Sara S. Mason, Colleen S. Albers, David Knopman, Kris Johnson, Rachelle S. Doody, Javier Villanueva Meyer, Munir Chowdhury, Susan Rountree, Mimi Dang, Yaakov Stern, Lawrence S. Honig, Karen L. Bell, Beau Ances, Maria Carroll, Sue Leon, Erin Householder, Mark A. Mintun, Stacy Schneider, Angela OliverNG, Randall Griffith, David Clark, David Geldmacher, John Brockington, Erik Roberson, Hillel Grossman, Effie Mitsis, Leyla deToledo-Morrell, Raj C. Shah, Ranjan Duara, Daniel Varon, Maria T. Greig, Peggy Roberts, Marilyn Albert, Chiadi Onyike, Daniel D.’ Agostino, Stephanie Kielb, James E. Galvin, Dana M. Pogorelec, Brittany Cerbone, Christina A. Michel, Henry Rusinek, Mony J. de Leon, Lidia Glodzik, Susan De Santi, P. Murali Doraiswamy, Jeffrey R. Petrella, Terence Z. Wong, Steven E. Arnold, Jason H. Karlawish, David A. Wolk, Charles D. Smith, Greg Jicha, Peter Hardy, Partha Sinha, Elizabeth Oates, Gary Conrad, Oscar L. Lopez, Mary Ann Oakley, Donna M. Simpson, Anton P. Porsteinsson, Bonnie S. Goldstein, Kim Martin, Kelly M. Makino, M. Saleem Ismail, Connie Brand, Ruth A. Mulnard, Gaby Thai, Catherine Mc Adams Ortiz, Kyle Womack, Dana Mathews, Mary Quiceno, Ramon Diaz Arrastia, Richard King, Myron Weiner, Kristen Martin Cook, Michael DeVous, Allan I. Levey, James J. Lah, Janet S. Cellar, Jeffrey M. Burns, Heather S. Anderson, Russell H. Swerdlow, Liana Apostolova, Kathleen Tingus, Ellen Woo, Daniel H. S. Silverman, Po H. Lu, George Bartzokis, Neill R. Graff Radford, Francine ParfittH, Tracy Kendall, Heather Johnson, Martin R. Farlow, Ann Marie Hake, Brandy R. Matthews, Scott Herring, Cynthia Hunt, Christopher H. van Dyck, Richard E. Carson, Martha G. MacAvoy, Howard Chertkow, Howard Bergman, Chris Hosein, Sandra Black, Bojana Stefanovic, Curtis Caldwell, Ging Yuek Robin Hsiung, Howard Feldman, Benita Mudge, Michele Assaly Past, Andrew Kertesz, John Rogers, Dick Trost, Charles Bernick, Donna Munic, Diana Kerwin, Marek Marsel Mesulam, Kristine Lipowski, Chuang Kuo Wu, Nancy Johnson, Carl Sadowsky, Walter Martinez, Teresa Villena, Raymond Scott Turner, Kathleen Johnson, Brigid Reynolds, Reisa A. Sperling, Keith A. Johnson, Gad Marshall, Meghan Frey, Jerome Yesavage, Joy L. Taylor, Barton Lane, Allyson Rosen, Jared Tinklenberg, Marwan N. Sabbagh, Christine M. Belden, Sandra A. Jacobson, Sherye A. Sirrel, Neil Kowall, Ronald Killiany, Andrew E. Budson, Alexander Norbash, Patricia Lynn Johnson, Thomas O. Obisesan, Saba Wolday, Joanne Allard, Alan Lerner, Paula Ogrocki, Leon Hudson, Evan Fletcher, Owen Carmichael, John Olichney, Charles DeCarli, Smita Kittur, Michael Borrie, T. Y. Lee, Rob Bartha, Sterling Johnson, Sanjay Asthana, Cynthia M. Carlsson, Steven G. Potkin, Adrian Preda, Dana Nguyen, Pierre Tariot, Adam Fleisher, Stephanie Reeder, Vernice Bates, Horacio Capote, Michelle Rainka, Douglas W. Scharre, Maria Kataki, Anahita Adeli, Earl A. Zimmerman, Dzintra Celmins, Alice D. Brown, Godfrey D. Pearlson, Karen Blank, Karen Anderson, Robert B. Santulli, Tamar J. Kitzmiller, Eben S. Schwartz, Kaycee M. SinkS, Jeff D. Williamson, Pradeep Garg, Franklin Watkins, Brian R. Ott, Henry Querfurth, Geoffrey Tremont, Stephen Salloway, Paul Malloy, Stephen Correia, Howard J. Rosen, Bruce L. Miller, Jacobo Mintzer, Kenneth Spicer, David Bachman, Elizabether Finger, Stephen Pasternak, Irina Rachinsky, John Rogers, Andrew Kertesz, Dick Drost, Nunzio Pomara, Raymundo Hernando, Antero Sarrael, Susan K. Schultz, Laura L. Boles Ponto, Hyungsub Shim, Karen Elizabeth Smith, Norman Relkin, Gloria Chaing, Lisa Raudin, Amanda Smith, Kristin Fargher, Balebail Ashok Raj, Australian Imaging Biomarkers and Lifestyle (AIBL) Study, Christopher Fowler, Stephanie R. Rainey-Smith, Sabine Bird, Julia Bomke, Pierrick Bourgeat, Belinda M. Brown, Samantha C. Burnham, Ashley I. Bush, Carolyn Chadunow, Steven Collins, James Doecke, Vincent Dore, Kathryn A. Ellis, Lis Evered, Amir Fazlollahi, Jurgen Fripp, Samantha L. Gardener, Simon Gibson, Robert Grenfell, Elise Harrison, Richard Head, Liang Jin, Adrian Kamer, Fiona Lamb, Nicola T. Lautenschlager, Simon M. Laws, Qiao-Xin Li, Lucy Lim, Yen Ying Lim, Andrea Louey, S. Lance Macaulay, Lucy Mackintosh, Ralph N. Martins, Paul Maruff, Colin L. Masters, Simon McBride, Lidija Milicic, Kelly Pertile, Tenielle Porter, Morgan Radler, Joanne Robertson, Mark Rodrigues, Christopher C. Rowe, Rebecca Rumble, Olivier Salvado, Greg Savage, Rosita Shishegar, Brendan Silbert, Magdalene Soh, Hamid R. Sohrabi, Kevin Taddei, Tania Taddei, Christine Thai, Brett Trounson, Regan Tyrrel, Michael Vacher, Shiji Varghese, Victor L. Villemagne, Michael Weinborn, Michael Woodward, Ying Xia, David Ames

**Affiliations:** 1 Centre for Precision Health, Edith Cowan University, 270 Joondalup Drive, Joondalup, Western Australia 6027 Australia; 2 Collaborative Genomics and Translation Group, School of Medical and Health Sciences, Edith Cowan University, Joondalup, Western Australia 6027 Australia; 3 CSIRO Health and Biosecurity, Australian E-Health Research Centre, Floreat, Western Australia 6014 Australia; 4 School of Pharmacy and Biomedical Sciences, Faculty of Health Sciences, Curtin Health Innovation Research Institute, Curtin University, Bentley, Western Australia 6102 Australia; 5 Australian E-Health Research Centre, CSIRO, Parkville, Victoria 3052 Australia; 6 Department of Molecular Imaging and Therapy and Centre for PET, Austin Health, Heidelberg, Victoria Australia; 7 Australian E-Health Research Centre, CSIRO, Herston, Queensland 4029 Australia; 8 School of Psychological Sciences and Turner Institute for Brain and Mental Health, Monash University, Melbourne, VIC Australia; 9 Department of Mathematics and Statistics, Curtin University, Bentley, Western Australia Australia; 10 School of Mathematics and Statistics, Murdoch University, Perth, Western Australia Australia; 11 Florey Institute of Neuroscience and Mental Health, The University of Melbourne, Parkville, VIC 3052 Australia; 12 Cogstate Ltd, Melbourne, VIC Australia; 13 Department of Psychiatry, University of Pittsburgh, Pittsburgh, PA USA

**Keywords:** DNA methylation, Epigenetics, Alzheimer’s disease, Hippocampal volume, Cognition, Ageing

## Abstract

The concept of age acceleration, the difference between biological age and chronological age, is of growing interest, particularly with respect to age-related disorders, such as Alzheimer’s Disease (AD). Whilst studies have reported associations with AD risk and related phenotypes, there remains a lack of consensus on these associations. Here we aimed to comprehensively investigate the relationship between five recognised measures of age acceleration, based on DNA methylation patterns (DNAm age), and cross-sectional and longitudinal cognition and AD-related neuroimaging phenotypes (volumetric MRI and Amyloid-β PET) in the Australian Imaging, Biomarkers and Lifestyle (AIBL) and the Alzheimer’s Disease Neuroimaging Initiative (ADNI). Significant associations were observed between age acceleration using the Hannum epigenetic clock and cross-sectional hippocampal volume in AIBL and replicated in ADNI. In AIBL, several other findings were observed cross-sectionally, including a significant association between hippocampal volume and the Hannum and Phenoage epigenetic clocks. Further, significant associations were also observed between hippocampal volume and the Zhang and Phenoage epigenetic clocks within Amyloid-β positive individuals. However, these were not validated within the ADNI cohort. No associations between age acceleration and other Alzheimer’s disease-related phenotypes, including measures of cognition or brain Amyloid-β burden, were observed, and there was no association with longitudinal change in any phenotype. This study presents a link between age acceleration, as determined using DNA methylation, and hippocampal volume that was statistically significant across two highly characterised cohorts. The results presented in this study contribute to a growing literature that supports the role of epigenetic modifications in ageing and AD-related phenotypes.

**Supplementary Information:**

The online version contains supplementary material available at 10.1007/s11357-022-00558-8.

## Introduction

Biological ageing, which affects most living organisms, can be characterised by a gradual loss of physical integrity, leading to impaired function and increased susceptibility to age-related disease and, ultimately, death [1]. Ageing is driven by genetic factors and external events, such as lifestyle, environment and their interaction [2]. Age is regarded as the most important non-modifiable risk factor for all neurodegenerative diseases, including Alzheimer’s disease (AD), and has been associated with changes in DNA methylation patterns [3].

Age-associated deregulation of the epigenome is a hallmark of the ageing process and has been studied extensively in recent years, which has resulted in evidence suggesting that changes in epigenetic patterns are dynamic through entire lifetimes in all species, tissues and cell types [4]. The ageing process leads to changes in DNA methylation patterns throughout the genome, and this has been termed epigenetic drift. Such changes result in genome wide hypomethylation and site-wide hypermethylation [2]. Epigenetic drift is unpredictable as it involves non-directional changes of both hypomethylation and hypermethylation of DNA. This limits any prediction of changes in the methylome amongst ageing individuals [2]. However, some evidence points towards the existence of ageing-associated differentially methylated regions (a-DMRs), which are consecutive groups of cytosine-phosphate-guanine (CpG) dinucleotides, sites that exhibit change in a constant direction over time [5–7]. Thus, methylation changes may not be purely stochastic but may also be associated with biological mechanisms closely linked to ageing processes and longevity.

DNA methylation clocks, also commonly referred to as epigenetic clocks, are DNA methylation-based estimates of biological age, which are developed through the combined use of mathematical algorithms and sets of CpGs that are strongly correlated with age (*r* ≥ 0.8) [8]. In 2011, Bocklandt et al. [9] developed an epigenetic clock that is able to predict chronological age in years, using peripheral blood with an average error of 5.2 years, based on 2 CpG sites present on the Illumina 27 k array. Of the epigenetic clocks developed since, the Hannum clock [10] was trained on blood derived DNA and comprises 71 CpG sites selected from the Illumina 450 k array, and the Horvath clock [11], developed around the same time, was constructed using multiple tissues and was intended to capture age-related changes, independent of tissue type. The Horvath clock is composed of 353 CpG sites that are all present on the earliest generation Illumina 27 k array [11]. Subsequently, Zhang and colleagues [12] developed two clocks based on two different training methods—best linear unbiased prediction (BLUP) and elastic net (EN). These two clocks were trained on a very large sample size of 13,661 samples, composed primarily of peripheral blood (13,402 samples) and saliva (259 samples). These clocks comprise 319,607 and 514 CpG sites, respectively, present on the Illumina 450 k and Illumina EPIC arrays. The second generation PhenoAge clock, developed by Levine and colleagues [13], is an epigenetic predictor of phenotypic age, a better representative than chronological age, of age-related biological dysregulation, derived from measures of clinical biomarkers. This difference led to substantial improvements in prediction of mortality and health span (number of years lived disease-free) compared to first generation clocks by Hannum and Horvath. The Phenoage clock was trained on blood derived DNA and comprises 513 CpG sites [13] and captures multifactorial ageing conditions, which is consistent with the fundamental underpinnings of ageing research.

Chronological age, defined as an individual’s legal age as calculated from birth to the current date, is not always an accurate indicator of the biological process of ageing, which makes it difficult to evaluate measures that promote longevity and healthy ageing [14]. Consequently, biological age has been proposed as a method to accurately predict the ageing status of an individual or tissue and could be reliably used to predict the onset of multiple diseases, assess disease risk and aid in the development of preventative strategies [14]. Since peripheral blood is easily accessible and largely non-invasive, it is suitable for multiple, repeated sampling over long periods of time, such as annual doctors’ visits, allowing for age or disease-related changes to be captured relatively early in the disease process and for appropriate preventative strategies based on the epigenetic evidence, to be put in place. Similarly, since obtaining samples is relatively non-invasive, multiple samples can be obtained during lifestyle interventions or drug trials, allowing response to treatment or intervention to be monitored easily and inexpensively, especially when compared to imaging modalities commonly used, such as magnetic resonance imaging (MRI) and positron emission topography (PET).

A measure of age acceleration (or deceleration) can be calculated based on the difference between an individual’s biological age (estimated through the use of epigenetic clocks) and chronological age (an individual’s legal age) [15]. Accelerated ageing has been documented in several genetic syndromes such as Down syndrome [16] and Werner’s syndrome [17]. Additionally, premature ageing in HIV infected individuals [18, 19] has been observed as well as in individuals with a high body mass index (BMI) and metabolic diseases [20]. As well, there is evidence for accelerated ageing in neurodegenerative diseases such as Parkinson’s disease [21], Huntington’s disease [22] and AD [15]. DNA methylation, both hyper- and hypomethylation, has been associated with AD in several brain regions [23–27]; however, it is still largely unclear whether markers in peripheral blood are truly reflective of the same changes as those observed in the brain [28]. In studies measuring DNA methylation age (DNAm age), accelerated ageing has been linked to an increase in AD pathology (diffuse plaques, neuritic plaques and Amyloid-β (Aβ) burden) [29], and in studies utilising peripheral DNAm age, accelerated ageing has been associated with reduced cognitive and physical fitness and an increase in all-cause mortality [15]. Whilst there is some evidence that accelerated ageing is associated with AD-related pathology and cognition, there is also conflicting research which does not support this. For example, Starnawska and colleagues [30] found that DNAm age is not associated with cognition in middle-age monozygotic twins and in a cohort of 964 middle-aged adults; Belsky et al. [31] also found no associations between DNAm age and cognition. However, it is difficult to compare results of studies as each utilised a different clock methodology to generate age estimates. Starnawska et al. [30] used the Hannum and Horvath clocks, and Belsky [31] used telomere length, the Klemera-Doubal method and pace of ageing clocks. Further, since the field is rapidly expanding and new clocks are being developed, consideration must be put into clock choice depending on the cohort and data available. Based on the inconsistency of results and the paucity of literature clearly describing the association of DNAm age and AD-related phenotypes, we set out to test the hypothesis that accelerated ageing is associated with differences in AD-related phenotypes. Using the highly characterised prospective longitudinal Australian Imaging, Biomarkers and Lifestyle (AIBL) study cohort we aimed to comprehensively investigate several methods of assessing DNAm age to assess (1) whether accelerated ageing is associated with cross-sectional measures of cognition and AD-related neuroimaging phenotypes (volumetric MRI and Aβ-PET) and (2) whether an individual’s current DNAm age is a predictor of future longitudinal changes in these two phenotypes. We then sought to test the robustness of our findings through validation within a similar highly characterised longitudinal cohort, the Alzheimer’s Disease Neuroimaging Initiative (ADNI).

## Materials and methods

### Participants

This study included participants enrolled in both the ongoing prospective longitudinal Australian Imaging, Biomarkers and Lifestyle and the multi-centre, longitudinal Alzheimer’s Disease Neuroimaging Initiative cohort studies. Detailed descriptions of both AIBL [32, 33] and ADNI [34] have been published previously. Participants enrolled in AIBL or ADNI were selected for inclusion in the current study only if methylation data and longitudinal (> 3 timepoints) data was available for the respective phenotype analysed (i.e. PET imaging and MRI or cognition).

### Neuroimaging and cognitive data

Individuals within the AIBL cohort underwent brain Aβ imaging by positron emission tomography (PET) using one of three tracers: ^11^C–Pittsburgh compound B (PiB), ^18^F-florbetapir or ^18^F-flutemetamol. Of these, 373 had > 3 timepoints and were included in this study. Similarly, ADNI participants underwent Aβ PET imaging studies with either ^18^F-florbetaben or ^18^F-florbetapir, with 486 participants included for validation purposes. In both cohorts, resulting Aβ PET scans were analysed using the CapAIBL software [35], an open access, web-based magnetic resonance (MR)-less algorithm, to generate standardised uptake value (SUV) ratios (SUVR) for all tracers and their associated. These tracer specific SUVR levels were then transformed and expressed in centiloid values (CL) as described previously [36, 37]. Aβ PET status was considered as Aβ negative (Aβ − ; < 20 CL) or Aβ positive (Aβ + ; ≥ 20 CL).

Of the 373 AIBL participants included in this study, 329 also had available MRI data, whilst 382 of the included ADNI participants underwent an MRI scan. MRI images were obtained at 3 T using the ADNI T1 magnetisation-prepared rapid gradient echo (MPRAGE) protocol with subsequent estimation of all cortical volumes from the T1 using Freesurfer, as previously described [13]. All volumes were corrected for normal ageing and ICV, with left and right volumes averaged. Volumetric corrections were made using a regression-based approach against a reference population that included ‘super’ healthy subjects, being cognitively unimpaired (MMSE > 28, CDR = 0) Ab negative individuals who did not carry an Apolipoprotein E (*APOE)* ε4 allele.

Both AIBL and ADNI participants undertook comprehensive neuropsychological assessment as previously described [32–34]. The resulting test scores were used for the calculation of the pre-Alzheimer’s cognitive composite (PACC), in AIBL, as described by Donohue et al. [38] and a modified PACC for ADNI [39]. This data was available at > 3 timepoints for 358 (out of 373) and 469 (out of 486) individuals from AIBL and ADNI, respectively.

### Genetic and epigenetic data

AIBL and ADNI study participant DNA was isolated for downstream analysis from whole blood using QIAamp DNA blood spin column kits (Qiagen, Valencia, CA, USA) as described previously [32–34]. Likewise, *APOE* genotyping protocols for AIBL and ADNI have been published previously [40, 41]. *APOE* carrier status was defined as the presence (one or two copies of the *APOE* ε4 allele) or absence (zero copies of the *APOE* ε4 allele).

DNA methylation analysis was conducted as previously described [41, 42]. Briefly, DNA samples were bisulphite converted using EZ DNA Methylation Kits (Zymo Research, Orange, CA, USA), and genome-wide DNA methylation patterns were analysed using the Infinium HumanMethylation EPIC (850 k) BeadChip array (Illumina, Inc., San Diego, CA, USA). BeadChips were washed, labelled using single-base extension, stained with multiple layers of fluorescence and scanned using the Illumina iScan system (Illumina Inc, CA). QC and normalisation of generated DNA methylation data were undertaken using the *meffil* package in R [43] (Version 3.5.0.) as previously described [41, 42]. Samples that failed QC were excluded from further analysis.

### Estimation of DNA methylation age

Five clock methodologies (Horvath [11], Hannum [10], Phenoage [13], Zhang elastic net (EN) and Zhang best linear unbiased prediction (BLUP)) [12] were utilised to calculate age estimates (DNAm age) for all AIBL (*n* = 373) and ADNI (*n* = 486) samples. Each clock is composed of a unique, defined set of CpG sites whose DNA methylation levels are used to generate an estimate of DNAm age. The CpG sites used in the calculation of each clock were chosen based on the statistical methodology specific to each clock [10–13]. In the current study, we utilised both disproportionate biological age (DBAge), which is the residual from regressing biological age on chronological age [29], and difference in age (DiffAge) [44], calculated by subtracting chronological age from biological age, as measures of age acceleration/deceleration. Both methodologies for calculating deviations in biological age from chronological age are a widely used and accepted methods to quantify ageing [44, 45]. The difference between DBAge and DiffAge is that the latter is a relative measure representing the difference between chronological age and biological age at the individual level, irrespective of other samples, whereas DBAge measures the difference between an individual’s DNAm age and the predicted DNAm age for that individual’s chronological age based on all samples present in the cohort. Thus, DiffAge measures the degree of ageing when compared to all other samples in the cohort. Here, we present only DBAge results as DiffAge and DBAge results were highly correlated in both the AIBL (Horvath *R*^2^ = 0.92; Zhang BLUP *R*^2^ = 0.97; Zhang EN *R*^2^ = 0.94, Phenoage *R*^2^ = 0.98; Supplementary Fig. 1, Additional File 1) and ADNI (Horvath *R*^2^ = 0.96; Zhang BLUP *R*^2^ = 1.00; Zhang EN *R*^2^ = 0.96, Phenoage *R*^2^ = 0.99; Supplementary Fig. 2, Additional File 1) cohorts. DiffAge results can be found in Additional file 1.

### Statistical analysis

Statistical analyses were carried out in R Version 4.1.2 for Macintosh. Baseline demographic data analyses provided means, standard deviations and percentages across the whole cohort and by confirmed classifications of cognitively unimpaired (CU), mild cognitive impairment (MCI) and Alzheimer’s disease (AD). Analysis of variance (ANOVA; age) and chi-squared tests (gender, years of education, *APOE* ε4 + ve, high Aβ burden, smoking status) were used to determine the significance of differences between groups. These demographic and clinical characteristics are summarised in Table 1. For all analyses described below, analyses were first undertaken in the AIBL sample, with associations surviving correction for false discovery rate (FDR) [46] subsequently tested in the ADNI sample.

**Table 1:** Baseline demographic information

AIBL	Whole cohort *n* = 373	Cognitively unimpaired *n* = 240	Mild cognitive impairment *n* = 69	Alzheimer’s disease *n* = 64	*p*
Age, mean (SD)	73.43 (6.99)	72.44 (6.42)	74.96 (7.58)	75.52 (7.74)	0.0003
Female *n* (%)	197 (52.8)	137 (57.1)	26 (37.7)	34 (53.1)	0.017
Years of education *n* (%)					
0–8	32 (8.57)	13 (5.4)	10 (14.5)	9 (14.1)	0.077
9–12	155 (41.55)	100 (41.7)	32 (46.4)	23 (35.9)	
13–15	75 (21.10)	53 (22.1)	9 (13.0)	13 (20.3)	
15 +	108 (28.95)	73 (30.4)	18 (26.1)	17 (26.6)	
* APOE* ε4 carriage *n* (%)	160 (42.89)	77 (32.08)	34 (49.27)	49 (76.56)	3.254e − 09
Αβ + *n* (%)	171 (45.84)	72 (30)	42 (60.86)	57 (89.06)	2.2e − 16
MRI *n* (%)	329 (88.2)	220 (91.66)	57 (82.60)	52 (81.25)	0.485
Smoking status *n* (%)	138 (36.99)	73 (30.41)	35 (50.72)	21 (32.81)	0.251
PACC mean (SD) [*n* (%)]	− 0.88 (1.33) [358 (95.97)]	− 0.18 (0.69) [239 (99.58)]	− 1.61 (0.83) [68 (98.55)]	− 3.26 (0.80) [51 (79.68)]	0.012
ADNI	Whole cohort *n* = 486	Cognitively unimpaired *n* = 166	Mild cognitive impairment *n* = 256	Alzheimer’s disease *n* = 64	*p*
Age, mean (SD)	73.92 (7.51)	75.91 (6.71)	72.05 (7.46)	76.26 (7.84)	0.09
Female *n* (%)	227 (46.70)	86 (51.80)	115 (44.92)	26 (40.62)	0.22
Years of education *n* (%)					
0–8	4 (0.8)	2 (1.20)	1 (0.40)	1 (1.60)	0.90
9–12	60 (12.30)	18 (10.84)	34 (13.28)	8 (12.50)	
13–15	89 (18.3)	31 (18.67)	45 (17.57)	13 (20.31)	
15 +	333 (68.5)	115 (69.27)	176 (68.75)	42 (65.62)	
* APOE* ε4 carriage *n* (%)	204 (41.97)	44 (26.50)	114 (44.53)	46 (71.87)	1.615e − 09
Αβ + *n* (%)	250 (51.4)	51 (30.72)	143 (55.85)	56 (87.50)	1.372e − 14
MRI *n* (%)	382 (78.60)	117 (79.48)	229 (89.45)	36 (56.25)	0.47
Smoking status *n* (%)	193 (39.7)	69 (41.60)	102 (39.84)	22 (34.37)	0.60
PACC mean (SD) [*n* (%)]	− 0.11 (0.53) [469 (96.50)]	0.10 (0.43) [164 (98.79)]	− 0.17 (0.51) [252 (98.43)]	− 0.54 (0.63) [53 (82.81)]	0.352

Baseline demographic and clinical characteristics of all participants with available methylation data in the AIBL study. *p* values represent significance when comparing between classifications

*Aβ* + high Aβ burden, *MRI* magnetic resonance imaging, *APOE ε4* apolipoprotein ε4 allele, *PACC* pre-Alzheimer’s cognitive composite

To determine whether accelerated age is associated with cross-sectional measures of cognition (the pre-Alzheimer’s cognitive composite (PACC)) and neuroimaging phenotypes (grey and white matter volume, hippocampal volume, ventricle volume and Aβ burden) in the brain, linear regressions were utilised. Phenotype outcomes (cognition and neuroimaging phenotypes) were set as the dependent variables, and the measures of methylation age estimates set as the independent variables. *APOE* ε4 (absence/presence), sex (binary), age (years), years of education (categorical) and smoking status [47] (binary) were included as covariates. The most appropriate model to fit the data was defined using a stepwise selection based on the Akaike information criterion (AIC) [48]; this model was defined as below:

$$\mathrm{Phenotype}\sim\mathrm{DiffAge}\;\mathrm{OR}\;\mathrm{DBAge}+\mathrm{Age}+\mathrm{Sex}+\mathrm{APOE}\varepsilon4+\mathrm{Years}\;\mathrm{of}\;\mathrm{education}+\mathrm{Smoking}\;\mathrm{Status}$$

To determine whether an individual’s current DBAge or DiffAge is an indicator of longitudinal change in cognition and neuroimaging phenotypes, linear regressions were utilised with FDR correction. Here, we calculated the rate of change in the outcome of interest (cognition and each neuroimaging phenotype), in individuals with at least three timepoints of assessments, using linear regressions to estimate individual model slopes. The slope value was then used as the dependent variable in subsequent analyses, with the model intercept included as a covariate, in addition to *APOE* ε4 (absence/presence), sex (binary), age (years), years of education (categorical) and smoking status (binary). As with previous model selection, the model with the best fit was chosen using the AIC and was defined as below:

$$\mathrm{Slope}\sim\mathrm{DiffAge}\;\mathrm{OR}\;\mathrm{DBAge}\;\mathrm{residual}+\mathrm{Intercept}+\mathrm{Age}+\mathrm{Sex}+\mathrm{APOE}\varepsilon4+\mathrm{Years}\;\mathrm{of}\;\mathrm{education}+\mathrm{Smoking}\;\mathrm{Status}$$

## Results

Demographic data for the AIBL and ADNI imaged cohorts and clinical classification (CU, MCI and AD) with available methylation data are presented in Table 1. This study assessed 373 AIBL participants (CU = 240, MCI = 60 and AD = 64), aged 73.43 ± 6.99 years with 197 females at baseline, and 486 ADNI participants (CU = 166, MCI = 256, AD = 64), aged 73.9 ± 7.51 years with 227 females at baseline. In the AIBL cohort, significant differences were observed when comparing age across clinical classifications (*p* = 0.0003, sex (*p* = 0.017), *APOE* ε4 allele carriage (*p* = 3.254e − 09) and high Aβ burden (*p* = 2.2e − 16). In the ADNI cohort, significant differences were observed when comparing *APOE* ε4 allele carriage (*p* = 1.615e − 09) and high Aβ burden (*p* = 1.372e − 14).

### Accelerated biological ageing is not associated with cross-section and longitudinal measures of cognition

In the cognitively unimpaired Aβ + cohort, a nominally significant association between cross-sectional PACC scores and accelerated ageing was observed with the Phenoage clock (Supplementary Table 1, Additional File 1). In the cognitively unimpaired Aβ − cohort, a nominally significant association between cross-sectional PACC scores and accelerated ageing was observed with the Horvath clock (Supplementary Table 1, Additional File 1). These associations did not remain significant after FDR correction. In the whole cohort, nominally significant associations were observed between change in PACC performance and accelerated ageing, using the Hannum Clock (Supplementary Table 6, Additional File 1). In the Aβ + cohort, nominally significant associations were observed between change in PACC performance and accelerated ageing, using the Hannum clock (Supplementary Table 6, Additional File 1). These associations did not remain significant after FDR correction.

### Accelerated biological ageing is not associated with cross-section and longitudinal measures of Aβ burden

No significant associations were observed between measures of age acceleration and Aβ burden, cross-sectionally, or longitudinally in the AIBL sample (Supplementary Tables 2 and 7, Additional File 1).

### Accelerated biological ageing is associated with cross-sectional measures of brain volume

In the whole cohort, nominally significant associations between hippocampal volume and accelerated ageing were observed with the Hannum and Phenoage clocks and between ventricle volume and the Hannum clock (Table 2). In the Aβ + cohort, nominally significant associations between hippocampal volume and accelerated ageing were observed with the Zhang EN, Hannum and Phenoage clocks (Table 2). In the Aβ − cohort, a nominally significant association between ventricle volume and accelerated ageing was observed with the Phenoage clock (Table 2). In the cognitively unimpaired cohort, nominally significant associations between white matter volume and hippocampal volume and accelerated ageing were observed with the Zhang BLUP and Hannum clocks (Table 2). In the cognitively unimpaired Aβ + cohort, nominally significant associations between hippocampal volume and accelerated ageing were observed with the Zhang EN, Hannum and Phenoage clocks (Table 2).

**Table 2:** AIBL cross-sectional hippocampal volume

Population (*n*)	Predictor	Estimate	SE	CI 95	*P* predictor
Whole cohort (329)	Zhang EN	− 0.021	0.013	− 0.047 – 0.005	0.149
	Zhang BLUP	− 0.009	0.013	− 0.034 – 0.016	0.471
	Hannum	− 0.029	0.009	− 0.047 – − 0.11	0.007
	Horvath	− 0.012	0.008	− 0.027 – 0.003	0.149
	**Phenoage**	** − 0.019**	**0.006**	** − 0.031 – − 0.006**	0.009**
Aβ + (145)	**Zhang EN**	** − 0.050**	**0.021**	** − 0.092 – − 0.008**	0.032**
	Zhang BLUP	− 0.030	0.020	− 0.070 – 0.010	0.136
	**Hannum**	** − 0.045**	**0.014**	** − 0.072 – − − 0.018**	0.003**
	Horvath	− 0.020	0.011	− 0.041 – 0.002	0.097
	**Phenoage**	** − 0.034**	**0.010**	** − 0.054 – − 0.015**	0.003**
Aβ − (184)	Zhang EN	0.002	0.015	− 0.028 – 0.032	0.875
	Zhang BLUP	0.014	0.015	− 0.016 – 0.044	0.875
	Hannum	− 0.009	0.011	− 0.031 – 0.014	0.875
	Horvath	0.002	0.010	− 0.017 – 0.022	0.875
	Phenoage	0.002	0.008	− 0.014 – 0.017	0.875
Cognitively unimpaired (220)	Zhang EN	− 0.014	0.014	− 0.041 – 0.013	0.507
	Zhang BLUP	− 0.001	0.014	− 0.028 – 0.025	0.921
	Hannum	− 0.024	0.010	− 0.044 – − 0.004	0.099
	Horvath	− 0.001	0.009	− 0.019 – 0.016	0.921
	Phenoage	− 0.010	0.007	− 0.024 – 0.004	0.414
Cognitively unimpaired Aβ + (65)	**Zhang EN**	** − 0.069**	**0.025**	** − 0.120 – − 0.019**	0.019**
	Zhang BLUP	− 0.034	0.024	− 0.082 – 0.015	0.209
	**Hannum**	** − 0.058**	**0.019**	** − 0.096 – − 0.020**	0.018**
	Horvath	− 0.013	0.018	− 0.049 – 0.023	0.483
	**Phenoage**	** − 0.038**	**0.016**	** − 0.069 – − 0.007**	0.030**
Cognitively unimpaired Aβ − (155)	Zhang EN	0.004	0.016	− 0.029 – 0.036	0.828
	Zhang BLUP	0.014	0.017	− 0.019 – 0.047	0.828
	Hannum	− 0.015	0.012	− 0.039 – 0.010	0.828
	Horvath	0.004	0.011	− 0.017 – 0.026	0.828
	Phenoage	− 0.004	0.008	− 0.020 – 0.012	0.828

AIBL cross-sectional results for associations between accelerated ageing (DBAge) and hippocampal volume. *p* values shown represent values after FDR correction. Bolded values with ** represent values that remain significant after FDR correction

*SE* standard error, *CI 95* 95% confidence intervals, *P predictor p* value of clock used, *EN* elastic net *BLUP* best linear unbiased prediction, *Aβ* Amyloid-β

After FDR correction, in the whole cohort, two associations remained significant, being associations between accelerated ageing and hippocampal volume using the Hannum clock (estimate =  − 0.029, SE = 0.009, CI =  − 0.047 −  − 0.110, *p* = 0.007; Table 2) and the Phenoage clocks (estimate =  − 0.019, SE = 0.006, CI =  − 0.031 −  − 0.006, *p* = 0.009; Table 2). After FDR correction, in the Aβ + cohort, associations between hippocampal volume and accelerated ageing using the Zhang EN (estimate =  − 0.050, SE = 0.021, CI =  − 0.092 −  − 0.008, *p* = 0.032, Table 2), Hannum (estimate =  − 0.045, SE = 0.014, CI =  − 0.072 −  − 0.018, *p* = 0.003; Table 2) and Phenoage (estimate =  − 0.034, SE = 0.010, CI =  − 0.054 −  − 0.015, *p* = 0.003; Table 2), clocks remained significant. In the cognitively unimpaired Aβ + cohort, associations between hippocampal volume and accelerated ageing using the Zhang EN (estimate =  − 0.069, SE = 0.025, CI =  − 0.120 −  − 0.019, *p* = 0.019; Table 2), Hannum (estimate =  − 0.058, SE = 0.019, CI =  − 0.096 −  − 0.020, *p* = 0.018; Table 2) and Phenoage (estimate =  − 0.038, SE = 0.016, CI =  − 0.069 −  − 0.007, p = 0.030; Table 2), clocks remained significant. This finding was validated within ADNI, in the cognitively unimpaired Aβ + cohort, where a significant association was observed between hippocampal volume and accelerated ageing, using the Hannum clock (estimate =  − 0.029, SE = 0.014, − 0.057 −  − 0.001, *p* = 0.046; Table 3). No other associations remained significant after FDR correction.

**Table 3:** ADNI cross-sectional hippocampal volume

Population (*n*)	Predictor	Estimate	SE	CI 95	*P* predictor
Whole cohort (382)	Zhang EN	− 0.004	0.006	− 0.016 – 0.007	0.482
	Zhang BLUP	− 0.004	0.006	− 0.015 – 0.007	0.445
	Hannum	− 0.002	0.004	− 0.011 – 0.006	0.602
	Horvath	− 0.003	0.004	− 0.010 – 0.004	0.425
	Phenoage	− 0.003	0.003	− 0.010 – 0.003	0.344
Aβ + (194)	Zhang EN	− 0.007	0.009	− 0.025 – 0.010	0.392
	Zhang BLUP	− 0.006	0.008	− 0.022 – 0.010	0.428
	Hannum	− 0.005	0.006	− 0.017 – 0.006	0.371
	Horvath	− 0.006	0.005	− 0.016 – 0.003	0.193
	Phenoage	− 0.007	0.005	− 0.016 – 0.002	0.146
Aβ − (188)	Zhang EN	− 0.001	0.008	− 0.017 – 0.015	0.866
	Zhang BLUP	− 0.001	0.008	− 0.016 – 0.014	0.862
	Hannum	0.005	0.006	− 0.008 – 0.017	0.471
	Horvath	0.000	0.005	− 0.010 – 0.011	0.955
	Phenoage	0.002	0.005	− 0.008 – 0.011	0.727
Cognitively unimpaired (117)	Zhang EN	− 0.004	0.008	− 0.019 – 0.011	0.568
	Zhang BLUP	− 0.008	0.007	− 0.021 – 0.006	0.264
	Hannum	− 0.013	0.006	− 0.025 – 0.000	0.043
	Horvath	0.000	0.005	− 0.009 – 0.010	0.920
	Phenoage	− 0.002	0.004	− 0.011 – 0.007	0.662
Cognitively unimpaired Aβ + (34)	Zhang EN	− 0.033	0.020	− 0.074 – 0.008	0.111
	Zhang BLUP	− 0.031	0.016	− 0.064 – 0.002	0.061
	**Hannum**	** − 0.029**	**0.014**	** − 0.057 – − 0.001**	0.046**
	Horvath	0.005	0.011	− 0.018 – 0.029	0.647
	Phenoage	− 0.013	0.011	− 0.036 – 0.011	0.268
Cognitively unimpaired Aβ − (83)	Zhang EN	− 0.002	0.009	− 0.019 – 0.016	0.825
	Zhang BLUP	− 0.002	0.008	− 0.018 – 0.013	0.770
	Hannum	− 0.011	0.008	− 0.027 – 0.004	0.143
	Horvath	− 0.004	0.006	− 0.015 – 0.008	0.544
	Phenoage	− 0.001	0.005	− 0.012 – 0.009	0.802

ADNI cross-sectional validation results for associations between accelerated ageing (DBAge) hippocampal volume. *p* values shown represent values before FDR correction. Bolded values with ** represent values that appeared significant

*SE* standard error, *CI 95* 95% confidence intervals, *P predictor p* value of clock used, *EN* elastic net, *BLUP* best linear unbiased prediction, *Aβ* Amyloid-β

### Accelerated biological ageing is associated with longitudinal measures of brain volume

In the whole cohort, nominally significant associations were observed between hippocampal volume (Supplementary Table 10) and ventricle volume (Supplementary Table 11, Additional File 1) and accelerated ageing, using the Hannum Clock. In the Aβ + cohort, nominally significant associations were observed between hippocampal volume and accelerated ageing, using the Hannum clock (Supplementary Table 10). In the cognitively unimpaired cohort, nominally significant associations were observed between grey matter volume and accelerated ageing, using the Hannum clock (Supplementary Table 10, Additional File 1). However, no significant associations remained after FDR correction.

## Discussion

This study aimed to comprehensively investigate several methods of ascertaining DNAm age to determine if accelerated ageing, calculated in two ways (DiffAge and DBAge), is associated with cross-sectional measures of cognition and AD-related neuroimaging phenotypes and if an individual’s current DNAm age is a predictor of longitudinal changes in the brain and cognition. We report no association of accelerated ageing with brain Aβ burden or measures of cognition and there was no evidence to support the hypothesis that an individual’s current DNAm age is a predictor of future changes in either cognition or neuroimaging phenotypes. However, accelerated ageing, when calculated using the Hannum and Phenoage clocks, was associated with cross-sectional measures of hippocampal volume, when assessed across all AIBL participants included. Further analyses showed that accelerated ageing, as determined using the Zhang EN, Hannum and Phenoage clocks, was also associated with hippocampal volume when limited to Aβ + individuals and likewise when this analysis was further limited to cognitively unimpaired Aβ + individuals. However, after validation in the ADNI cohort, significant associations between accelerated ageing and hippocampal volume were limited to those derived from the Hannum clock only. In cognitively unimpaired individuals with high brain Aβ burden, a smaller hippocampal volume was observed in individuals with a larger deviation of biological age from chronological age in both in the AIBL and the ADNI cohort. This relationship may be driven by an elevated brain Aβ burden (Aβ positive) in combination with an advanced biological age and explains why this relationship is not observed in the Aβ negative cohorts. There is some evidence to substantiate this by Levine et al. [29], in their study in which there was an association with age acceleration and Aβ load. However, it should be noted that this study was performed on post-mortem pre-frontal cortex brain tissue and the results cannot be directly compared. Further research is therefore needed to examine this relationship in more detail.

Whilst not observed in the present study, longitudinal associations between decline in cognition and age acceleration have been previously observed. Results from the Betula study in Sweden demonstrated that episodic memory performance over 15 years was maintained in ageing in those individuals with a lower DNAm age (calculated using the Horvath clock) [49]. This study included a small sample size of 52 participants and a low participant baseline age (55–65 years), both of which may account for the difference in their findings. In a twin study investigating the relationship between age acceleration and cognitive impairment, Vaccarino et al. [50] showed a faster rate of decline in cognition in individuals who had an older DNAm age (calculated using the Horvath clock) relative to their twin, over an average of 11.5 years. Moreover, this study only included men, and it has been demonstrated that men have a greater age acceleration than women [51]. In a recent study, Beydoun et al. [52] found an association between accelerated age and decline in attention and visuospatial/visuoconstruction ability, in men but not women, using the Hannum clock. In contrast to the study presented here, which focussed on the PACC as a measure of global cognitive decline, Beydoun et al. [52] and Degerman et al. [49] used domain specific measures which may not be directly comparable. Additionally, a constraint of their analysis, and likely their findings, was the inclusion of two timepoints for the cognitive outcomes of interest, and though they report significant findings, these could be due to random variations in cognitive performance rather than a meaningful decline over time [52]. In the Lothian Birth Cohort, age acceleration (calculated using the Horvath clock) was associated with cross-sectional measures of lower cognition (general fluid type intelligence derived from the Wechsler Adult Intelligence Scale-III^UK^), weaker grip strength and poorer lung function [53]. A lack of longitudinal association may have been caused by the relatively short follow-up time (6 years), where only small changes in cognition occurred [53].

Similar to studies assessing relationships with cognition, there is also a lack of consistency across studies with respect to neuroimaging phenotypes. Further, there is very limited research with regard to the association of accelerated ageing and neuroimaging phenotypes. To our knowledge, this is the first study to uncover an association between age acceleration and reduced hippocampal volume and specifically only in preclinical AD and not with ageing in the absence of disease (brain Aβ). A small number of other studies have investigated the association of age acceleration and neuroimaging measures with varied results. Hodgson et al. [54] observed that with increasing age acceleration (calculated using the Horvath clock), white matter integrity, both locally and within specific regions of the brain, decreased. Similarly, Hillary et al. [55] observed that higher DNAm age (calculated using the GrimAge clock) was significantly associated with decreased overall brain volume (white and grey matter) and increased white matter hyperintensities. In the current study, nominally significant associations between age acceleration and an increase in ventricle volume were observed in the AIBL cohort, which is indicative of an overall smaller brain volume. Levine et al. [29] demonstrated that age acceleration was associated with diffuse plaques, neuritic plaques, Aβ burden and a trend towards an association with neurofibrillary tangles. Chouliaras et al. [56] utilised the Whitehall II imaging sub-study and observed a significant association between accelerated age (calculated using the Hannum clock) and MRI measures; global measure of fractional anisotropy and decreased mean diffusivity, which appeared to be in the opposite direction of similar studies [57]. As is evident, it is hard to compare the results of studies that investigate associations with age acceleration due to a lack of consistency in the availability of data between cohorts, as well as study design, outcomes of interest and clock choice. As such, very few findings have been replicated across more than one study, which increases the potential of false positive findings being published [58].

Finally, the limited findings across studies might be reflective of the limitations of the existing clocks themselves. It should be noted that whilst epigenetic clocks are good at predicting age, there is some evidence to suggest that bespoke clocks that are more disease and/or outcome/phenotype-specific would be better suited for assessing pathological changes and disease progression in unavailable tissue, such as the brain [59, 60]. For example, Grodstein et al. compared the performance of an epigenetic clock trained in cortex to clocks trained in blood, with stronger associations present across all outcomes of interest in the clock trained in brain tissue [60]. Similarly, Porter et al. demonstrated that in clocks trained in specific tissues, the CpG sites included often lead to poor predictive capabilities in other tissues [59]. However, one of the overarching aims of the current study was to assess if markers in peripheral blood are truly reflective of the same changes as those which are observed in the brain and if blood has the potential to be utilised as a surrogate tissue, as obtaining the tissue of interest, regardless of its performance, is not always feasible. The results presented in this study are robust and provide evidence that supports the role of epigenetic clocks in identifying AD-related phenotypes; however, further research, for example into the efficacy of phenotypic specific epigenetic ‘clocks’ or profiles, is warranted.

## Limitations

A limitation of this study is that our age acceleration measures were derived from DNA extracted from whole blood and not brain tissue. However, it has previously been demonstrated that age-related DNA changes are conserved across tissue and cell types [11, 61]. Additionally, it is evidenced in several disorders including Huntington’s disease [22], Down syndrome [16] and HIV infections [19] that age acceleration can be observed in both blood and brain tissue. Furthermore, there is a strong correlation between epigenetic profiles of different tissues sampled from the same individual, with the observed correlation between blood and brain methylation being higher than the correlation for gene expression [11, 61]. This study was performed in the AIBL cohort and validated in the ADNI cohort, both of which are representative of a predominantly Caucasian population. As such, these findings should be replicated in ethnically diverse cohorts to determine whether the methods are applicable to more generalised populations. As well, due to the voluntary nature of the AIBL and ADNI studies, the outcome are cohorts that are highly educated, and any observations made here might not be observed in general populations. Additionally, within the cohorts, there is an overrepresentation of samples collected in the later stages of life, and with this, there is a relatively narrow age range at the higher age spectrum. This has previously been observed to potentially influence the calculation of age estimates, specifically through the underestimation of age [62–64]. Even though the EPIC chip is currently the most comprehensive array chip available, it only assesses ~ 3% of CpG sites within the genome, it is possible that the CpGs present do not probe some of the most biologically informative sites. As well, the more biologically informative CpG sites may have not been selected by the modelling algorithms as they did not correlate well with chronological age.

## Conclusion

This study is one of only a few which has examined cross-sectional and longitudinal changes in cognitive function and the neuroimaging phenotypes of volumetric MRI and Amyloid-β PET as a function of age acceleration. Further, it is the first to assess and compare multiple methodologies for the calculation of age acceleration in two well-characterised longitudinal ageing cohorts. Although we were only able to identify an association of age acceleration with cross-sectional hippocampal volume, our study is strengthened by the use of a comprehensive set of epigenetic clocks, derived using the best genome-wide DNA methylation array currently available. Our results contribute to a growing literature that supports the role of epigenetic modifications in ageing and Alzheimer’s disease-related phenotypes. Due to their potentially reversible nature, epigenetic modifications may provide a powerful means for a therapeutic target and prevention and intervention strategies in ageing and age-related diseases, such as Alzheimer’s disease.

## Supplementary Information

Below is the link to the electronic supplementary material.

Supplementary file1 (DOCX 418 KB) Supplementary DBAge tables of non-significant results and supplementary DiffAge tables of data not included in manuscript due to high correlation with DBAge. Supplementary scatterplot figures of correlation of DBAge and DiffAge for each of the five clocks used in analyses.

## Data Availability

All AIBL data, and that specific to this study, is publicly accessible to all interested parties through an Expression of Interest procedure and is governed by the AIBL Data Use Agreement (aibl.csiro.au/awd). AIBL DNAm data are available from the GEO repository accession number GSE153712. All data derived from the ADNI cohort, and that specific to this study, are available to researchers by request as outlined in the ADNI access policy (adni.loni.usc.edu).

## Abbreviations

AD: Alzheimer’s disease
DNAm age: DNA methylation age
AIBL: Australian Imaging Biomarkers and Lifestyle
ADNI: Alzheimer’s Disease Neuroimaging Initiative
Aβ: Amyloid-β
a-DMRs: Ageing-associated differentially methylated regions
CpG: Cytosine-phosphate-guanine
BLUP: Best linear unbiased prediction
EN: Elastic net
MRI: Magnetic resonance imaging
PET: Positron emission topography
HIV: Human immunodeficiency virus
BMI: Body mass index
SUV: Standardised uptake value
SUVR: Standardised uptake value ratio
CL: Centiloids
MPRAGE: Magnetisation-prepared rapid gradient echo
ICV: Intra-cranial volume
MMSE: Mini-mental state exam
CDR: Clinical dementia rating
APOE: Apolipoprotein E
PACC: Pre-Alzheimer’s cognitive composite
PiB: ^11^C–Pittsburgh compound B
DiffAge: Difference in age
DBAge: Disproportionate biological ageing
MCI: Mild cognitive impairment
CU: Cognitively unimpaired
AIC: Akaike information criterion
FDR: False discovery rate
CI: Confidence interval
SE: Standard error
ANOVA: Analysis of variance

## References

[1] López-Otín C, Blasco MA, Partridge L, Serrano M, Kroemer G (2013). The hallmarks of aging. Cell.

[2] Zampieri M, Ciccarone F, Calabrese R, Franceschi C, Bürkle A, Caiafa P (2015). Reconfiguration of DNA methylation in aging. Mech Ageing Dev.

[3] Xiao F-H, Kong Q-P, Perry B, He Y-H (2016). Progress on the role of DNA methylation in aging and longevity. Brief Funct Genom.

[4] Christiansen L, Lenart A, Tan Q, Vaupel JW, Aviv A, McGue M (2016). DNA methylation age is associated with mortality in a longitudinal Danish twin study. Aging Cell.

[5] Bell CG, Xia Y, Yuan W, Gao F, Ward K, Roos L (2016). Novel regional age-associated DNA methylation changes within human common disease-associated loci. Genome Biol.

[6] Rakyan VK, Down T, Maslau S, Andrew T, Yang T-P, Beyan H, et al. Human aging-associated DNA hypermethylation occurs preferentially at bivalent chromatin domains. Genome Res. 2010;20(4):434–9. 10.1101/gr.103101.109 PMC2847746 20219945

[7] Spiers H, Hannon E, Wells S, Williams B, Fernandes C, Mill J (2016). Age-associated changes in DNA methylation across multiple tissues in an inbred mouse model. Mech Ageing Dev.

[8] Bell CG, Lowe R, Adams PD, Baccarelli AA, Beck S, Bell JT (2019). DNA methylation aging clocks: challenges and recommendations. Genome Biol.

[9] Bocklandt S, Lin W, Sehl ME, Sánchez FJ, Sinsheimer JS, Horvath S (2011). Epigenetic predictor of age. PLoS ONE.

[10] Hannum G, Guinney J, Zhao L, Zhang L, Hughes G, Sadda S (2013). Genome-wide methylation profiles reveal quantitative views of human aging rates. Mol Cell.

[11] Horvath S (2013). DNA methylation age of human tissues and cell types. Genome Biol.

[12] Zhang Q, Vallerga CL, Walker RM, Lin T, Henders AK, Montgomery GW (2019). Improved precision of epigenetic clock estimates across tissues and its implication for biological ageing. Genome Med.

[13] Levine ME, Lu AT, Quach A, Chen BH, Assimes TL, Bandinelli S (2018). An epigenetic biomarker of aging for lifespan and healthspan. Aging.

[14] Jiang S, Guo Y (2020). Epigenetic clock: DNA methylation in aging. Stem Cells Int.

[15] McCartney DL, Stevenson AJ, Walker RM, Gibson J, Morris SW, Campbell A (2018). Investigating the relationship between DNA methylation age acceleration and risk factors for Alzheimer’s disease. Alzheimers Dement Diagn Assess Dis Monit.

[16] Horvath S, Garagnani P, Bacalini MG, Pirazzini C, Salvioli S, Gentilini D (2015). Accelerated epigenetic aging in Down syndrome. Aging Cell.

[17] Maierhofer A, Flunkert J, Oshima J, Martin GM, Haaf T, Horvath S (2017). Accelerated epigenetic aging in Werner syndrome. Aging.

[18] Gross AM, Jaeger PA, Kreisberg JF, Licon K, Jepsen KL, Khosroheidari M (2016). Methylome-wide analysis of chronic HIV infection reveals five-year increase in biological age and epigenetic targeting of HLA. Mol Cell.

[19] Horvath S, Levine AJ (2015). HIV-1 infection accelerates age according to the epigenetic clock. J Infect Dis.

[20] Quach A, Levine ME, Tanaka T, Lu AT, Chen BH, Ferrucci L (2017). Epigenetic clock analysis of diet, exercise, education, and lifestyle factors. Aging.

[21] Horvath S, Ritz BR (2015). Increased epigenetic age and granulocyte counts in the blood of Parkinson’s disease patients. Aging.

[22] Horvath S, Langfelder P, Kwak S, Aaronson J, Rosinski J, Vogt TF (2016). Huntington’s disease accelerates epigenetic aging of human brain and disrupts DNA methylation levels. Aging.

[23] Roubroeks JA, Smith RG, van den Hove DL, Lunnon K (2017). Epigenetics and DNA methylomic profiling in Alzheimer’s disease and other neurodegenerative diseases. J Neurochem.

[24] De Jager PL, Srivastava G, Lunnon K, Burgess J, Schalkwyk LC, Yu L (2014). Alzheimer’s disease: early alterations in brain DNA methylation at ANK1, BIN1, RHBDF2 and other loci. Nat Neurosci.

[25] Lunnon K, Smith R, Hannon E, De Jager PL, Srivastava G, Volta M (2014). Methylomic profiling implicates cortical deregulation of ANK1 in Alzheimer’s disease. Nat Neurosci.

[26] Watson CT, Roussos P, Garg P, Ho DJ, Azam N, Katsel PL (2016). Genome-wide12 DNA methylation profiling in the superior temporal gyrus reveals epigenetic signatures associated with Alzheimer’s disease. Genome Med.

[27] Zhao J, Zhu Y, Yang J, Li L, Wu H, De Jager PL (2017). A genome-wide profiling of brain DNA hydroxymethylation in Alzheimer’s disease. Alzheimers Dement.

[28] Fransquet PD, Lacaze P, Saffery R, McNeil J, Woods R, Ryan J (2018). Blood DNA methylation as a potential biomarker of dementia: a systematic review. Alzheimers Dement.

[29] Levine ME, Lu AT, Bennett DA, Horvath S (2015). Epigenetic age of the pre-frontal cortex is associated with neuritic plaques, amyloid load, and Alzheimer’s disease related cognitive functioning. Aging.

[30] Starnawska A, Tan Q, Lenart A, McGue M, Mors O, Børglum AD (2017). Blood DNA methylation age is not associated with cognitive functioning in middle-aged monozygotic twins. Neurobiol Aging.

[31] Belsky DW, Moffitt TE, Cohen AA, Corcoran DL, Levine ME, Prinz JA (2018). Eleven telomere, epigenetic clock, and biomarker-composite quantifications of biological aging: do they measure the same thing?. Am J Epidemiol.

[32] Ellis KA, Bush AI, Darby D, De Fazio D, Foster J, Hudson P (2009). The Australian Imaging, Biomarkers and Lifestyle (AIBL) study of aging: methodology and baseline characteristics of 1112 individuals recruited for a longitudinal study of Alzheimer’s disease. Int Psychogeriatr.

[33] Fowler C, Rainey-Smith SR, Bird S, Bomke J, Bourgeat P, Brown BM (2021). Fifteen years of the Australian Imaging, Biomarkers and Lifestyle (AIBL) Study: progress and observations from 2,359 older adults spanning the spectrum from cognitive normality to Alzheimer’s disease. J Alzheimers Dis Rep.

[34] Mueller SG, Weiner MW, Thal LJ, Petersen RC, Jack C, Jagust W (2005). The Alzheimer’s disease neuroimaging initiative. Neuroimaging Clin N Am.

[35] Bourgeat P, Villemagne VL, Dore V, Brown B, Macaulay SL, Martins R (2015). Comparison of MR-less PiB SUVR quantification methods. Neurobiol Aging.

[36] Bourgeat P, Doré V, Fripp J, Ames D, Masters CL, Salvado O (2018). Implementing the centiloid transformation for 11C-PiB and β-amyloid 18F-PET tracers using CapAIBL. Neuroimage.

[37] Su Y, Flores S, Hornbeck RC, Speidel B, Vlassenko AG, Gordon BA (2018). Utilizing the Centiloid scale in cross-sectional and longitudinal PiB PET studies. Neuro Image Clin.

[38] Donohue MC, Sperling RA, Salmon DP, Rentz DM, Raman R, Thomas RG (2014). The preclinical Alzheimer cognitive composite: measuring amyloid-related decline. JAMA Neurol.

[39] Insel PS, Weiner M, Mackin RS, Mormino E, Lim YY, Stomrud E (2019). Determining clinically meaningful decline in preclinical Alzheimer disease. Neurology.

[40] Porter T, Villemagne VL, Savage G, Milicic L, Ying Lim Y, Maruff P (2018). Cognitive gene risk profile for the prediction of cognitive decline in presymptomatic Alzheimer’s disease. Pers Med Psych.

[41] Petersen RC, Aisen PS, Beckett LA, Donohue MC, Gamst AC, Harvey DJ (2010). Alzheimer's Disease Neuroimaging Initiative (ADNI). Neurology.

[42] Nabais MF, Laws SM, Lin T, Vallerga CL, Armstrong NJ, Blair IP (2021). Meta-analysis of genome-wide DNA methylation identifies shared associations across neurodegenerative disorders. Genome Biol.

[43] Min JL, Hemani G, Davey Smith G, Relton C, Suderman M (2018). Meffil: efficient normalization and analysis of very large DNA methylation datasets. Bioinformatics.

[44] Chen BH, Marioni RE, Colicino E, Peters MJ, Ward-Caviness CK, Tsai P-C (2016). DNA methylation-based measures of biological age: meta-analysis predicting time to death. Aging.

[45] Declerck K, Vanden BW (2018). Back to the future: epigenetic clock plasticity towards healthy aging. Mech Ageing Dev.

[46] Benjamini Y, Hochberg Y (1995). Controlling the false discovery rate: a practical and powerful approach to multiple testing. J Roy Stat Soc: Ser B (Methodol).

[47] Lee KWK, Pausova Z (2013). Cigarette smoking and DNA methylation. Front Genet.

[48] Akaike H, Parzen E, Tanabe K, Kitagawa G (1998). A new look at the statistical model identification. Selected Papers of Hirotugu Akaike.

[49] Degerman S, Josefsson M, Nordin Adolfsson A, Wennstedt S, Landfors M, Haider Z (2017). Maintained memory in aging is associated with young epigenetic age. Neurobiol Aging.

[50] Vaccarino V, Huang M, Wang Z, Hui Q, Shah AJ, Goldberg J, et al. Epigenetic age acceleration and cognitive decline: a twin study. J Gerontol A Biol Sci Med Sci. 2021;76(10):1854–63. 10.1093/gerona/glab047 PMC8436988 33606025

[51] Horvath S, Gurven M, Levine ME, Trumble BC, Kaplan H, Allayee H (2016). An epigenetic clock analysis of race/ethnicity, sex, and coronary heart disease. Genome Biol.

[52] Beydoun MA, Shaked D, Tajuddin SM, Weiss J, Evans MK, Zonderman AB (2020). Accelerated epigenetic age and cognitive decline among urban-dwelling adults. Neurology.

[53] Marioni RE, Shah S, McRae AF, Ritchie SJ, Muniz-Terrera G, Harris SE (2015). The epigenetic clock is correlated with physical and cognitive fitness in the Lothian Birth Cohort 1936. Int J Epidemiol.

[54] Hodgson K, Carless MA, Kulkarni H, Curran JE, Sprooten E, Knowles EE (2017). Epigenetic age acceleration assessed with human white-matter images. J Neurosci.

[55] Hillary RF, Stevenson AJ, Cox SR, McCartney DL, Harris SE, Seeboth A, et al. An epigenetic predictor of death captures multi-modal measures of brain health. Mol Psychiatry. 2021;26(8):3806–16. 10.1038/s41380-019-0616-9 PMC8550950 31796892

[56] Chouliaras L, Pishva E, Haapakoski R, Zsoldos E, Mahmood A, Filippini N (2018). Peripheral DNA methylation, cognitive decline and brain aging: pilot findings from the Whitehall II imaging study. Epigenomics.

[57] Mak E, Gabel S, Mirette H, Su L, Williams GB, Waldman A (2017). Structural neuroimaging in preclinical dementia: from microstructural deficits and grey matter atrophy to macroscale connectomic changes. Ageing Res Rev.

[58] Fransquet PD, Ryan J (2019). The current status of blood epigenetic biomarkers for dementia. Crit Rev Clin Lab Sci.

[59] Porter HL, Brown CA, Roopnarinesingh X, Giles CB, Georgescu C, Freeman WM (2021). Many chronological aging clocks can be found throughout the epigenome: implications for quantifying biological aging. Aging Cell.

[60] Grodstein F, Lemos B, Yu L, Klein H-U, Iatrou A, Buchman AS (2021). The association of epigenetic clocks in brain tissue with brain pathologies and common aging phenotypes. Neurobiol Dis.

[61] Horvath S, Zhang Y, Langfelder P, Kahn RS, Boks MPM, van Eijk K (2012). Aging effects on DNA methylation modules in human brain and blood tissue. Genome Biol.

[62] El Khoury LY, Gorrie-Stone T, Smart M, Hughes A, Bao Y, Andrayas A (2019). Systematic underestimation of the epigenetic clock and age acceleration in older subjects. Genome Biol.

[63] Shireby GL, Davies JP, Francis PT, Burrage J, Walker EM, Neilson GWA (2020). Recalibrating the epigenetic clock: implications for assessing biological age in the human cortex. Brain.

[64] Dhingra R, Kwee LC, Diaz-Sanchez D, Devlin RB, Cascio W, Hauser ER (2019). Evaluating DNA methylation age on the Illumina MethylationEPIC Bead Chip. PLoS ONE.

