# Supplementary material for: Comprehensive analysis of epigenetic clocks reveals associations between disproportionate biological ageing and hippocampal volume
Source: GeroScience. 2022 Apr 21;44(3):1807–23. doi: 10.1007/s11357-022-00558-8 (PMC9213584; doi:10.1007/s11357-022-00558-8)
Supplement: Supplementary file 1 — Supplementary file1 (DOCX 418 KB) Supplementary DBAge tables of non-significant results and supplementary DiffAge tables of data not included in manuscript due to high correlation with DBAge. Supplementary scatterplot figures of correlation of DBAge and DiffAge for each of the five clocks used in analyses. [file 11357_2022_558_MOESM1_ESM.docx]

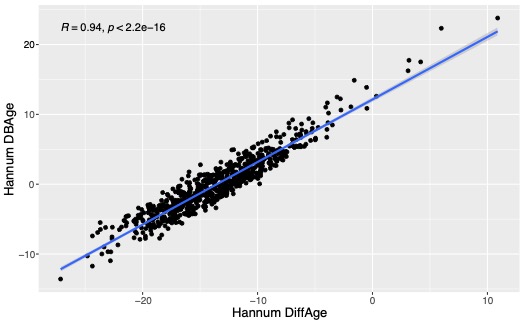
Supplementary Figure 1. Scatterplot of DiffAge (x axis) and DBAge (y axis) in AIBL calculated using the Hannum Clock.

*R* = 0.94, *p* < 2.2e-16.


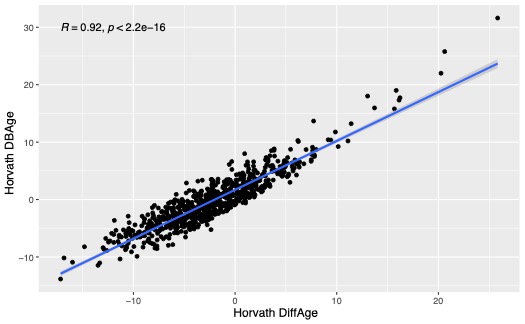


Supplementary Figure 2. Scatterplot of DiffAge (x axis) and DBAge (y axis) in AIBL calculated using the Horvath Clock.

*R* = 0.92, *p* < 2.2e-16.

Supplementary Table 1. AIBL Cross-Sectional PACC

| ­Population (n) | Predictor | Estimate | SE | CI 95 | *P* Predictor |
| --- | --- | --- | --- | --- | --- |
| Whole Cohort (358) | Zhang EN | 0.038 | 0.022 | -0.005 – 0.083 | 0.296 |
|  | Zhang BLUP | 0.025 | 0.022 | -0.017 – 0.068  0.068 | 0.392 |
|  | Hannum | 0.001 | 0.015 | -0.027 – 0.031 | 0.999 |
|  | Horvath | 0.020 | 0.013 | -0.005 – 0.046 | 0.269 |
|  | Phenoage | 0.000 | 0.011 | -0.022 – 0.022 | 0.999 |
| Aβ+ (159) | Zhang EN | 0.019 | 0.038 | -0.057 – 0.094 | 0.865 |
|  | Zhang BLUP | -0.001 | 0.036 | -0.073 – 0.070  -0.050.039 | 0.970 |
|  | Hannum | -0.010 | 0.025 | -0.059 – 0.039 | 0.865 |
|  | Horvath | 0.011 | 0.021 | -0.030 – 0.052 | 0.865 |
|  | Phenoage | 0.010 | 0.019 | -0.027 – 0.048 | 0.865 |
| Aβ- (199) | Zhang EN | 0.041 | 0.021 | -0.001 – 0.083 | 0.171 |
|  | Zhang BLUP | 0.038 | 0.021 | -0.003 – 0.079 | 0.171 |
|  | Hannum | 0.007 | 0.014 | -0.021 – 0.035 | 0.774 |
|  | Horvath | 0.022 | 0.013 | -0.004 – 0.049 | 0.171 |
|  | Phenoage | 0.001 | 0.011 | -0.020 – 0.021 | 0.954 |
| Cognitively Unimpaired (239) | Zhang EN | 0.026 | 0.015 | -0.003 – 0.056 | 0.139 |
|  | Zhang BLUP | 0.028 | 0.015 | -0.001 – 0.056 | 0.139 |
|  | Hannum | 0.008 | 0.011 | -0.013 – 0.028 | 0.474 |
|  | Horvath | 0.019 | 0.010 | 0.000 – 0.038 | 0.139 |
|  | Phenoage | 0.007 | 0.008 | -0.008 – 0.023 | 0.422 |
| Cognitively Unimpaired Aβ+ (72) | Zhang EN | 0.038 | 0.030 | -0.022 – 0.098 | 0.344 |
|  | Zhang BLUP | 0.040 | 0.027 | -0.014 – 0.093 | 0.344 |
|  | Hannum | 0.022 | 0.022 | -0.023 – 0.066 | 0.423 |
|  | Horvath | 0.010 | 0.020 | -0.030 – 0.050 | 0.628 |
|  | Phenoage | 0.040 | 0.016 | 0.008 – 0.072 | 0.078 |
| Cognitively Unimpaired Aβ- (167) | Zhang EN | 0.022 | 0.018 | -0.013 – 0.057 | 0.463 |
|  | Zhang BLUP | 0.019 | 0.018 | -0.016 – 0.054 | 0.463 |
|  | Hannum | 0.005 | 0.012 | -0.020 – 0.029 | 0.780 |
|  | Horvath | 0.023 | 0.011 | 0.001 – 0.046 | 0.197 |
|  | Phenoage | 0.003 | 0.009 | -0.015 – 0.020 | 0.780 |

Supplementary Table 1. AIBL cross-sectional results for associations between accelerated ageing (DBAge) and PACC. *P* values shown represent values after FDR correction. Bolded values with ** represent values that remain significant after FDR correction. SE, standard error, CI 95, 95% confidence intervals, P Predictor, *p* value of clock used, EN Elastic Net, BLUP Best Linear Unbiased Prediction.

Supplementary Table 6. AIBL Longitudinal PACC

| ­Population (n) | Predictor | Estimate | SE | CI 95 | *P* Predictor |
| --- | --- | --- | --- | --- | --- |
| Whole Cohort (358) | Zhang EN | -0.003 | 0.005 | -0.012 – 0.006 | 0.504 |
|  | Zhang BLUP | -0.004 | 0.005 | -0.013 – 0.005 | 0.429 |
|  | Hannum | -0.006 | 0.003 | -0.012 – 0.000 | 0.223 |
|  | Horvath | -0.004 | 0.003 | -0.009 – 0.002 | 0.429 |
|  | Phenoage | -0.002 | 0.002 | -0.007 – 0.002 | 0.429 |
| Aβ+ (159) | Zhang EN | -0.008 | 0.011 | -0.031 – 0.015 | 0.604 |
|  | Zhang BLUP | -0.021 | 0.011 | -0.043 – 0.001 | 0.148 |
|  | Hannum | -0.015 | 0.008 | -0.031 – 0.000 | 0.148 |
|  | Horvath | -0.007 | 0.008 | -0.023 – 0.009 | 0.604 |
|  | Phenoage | -0.002 | 0.006 | -0.015 – 0.011 | 0.743 |
| Aβ- (199) | Zhang EN | 0.000 | 0.004 | -0.008 – 0.007 | 0.940 |
|  | Zhang BLUP | 0.004 | 0.004 | -0.003 – 0.012 | 0.917 |
|  | Hannum | -0.002 | 0.003 | -0.007 – 0.003 | 0.917 |
|  | Horvath | -0.001 | 0.002 | -0.005 – 0.004 | 0.940 |
|  | Phenoage | -0.001 | 0.002 | -0.004 – 0.003 | 0.940 |
| Cognitively Unimpaired (239) | Zhang EN | 0.004 | 0.004 | -0.003 – 0.011 | 0.526 |
|  | Zhang BLUP | 0.005 | 0.004 | -0.002 – 0.012 | 0.526 |
|  | Hannum | -0.002 | 0.002 | -0.007 – 0.002 | 0.526 |
|  | Horvath | 0.000 | 0.002 | -0.005 – 0.004 | 0.856 |
|  | Phenoage | -0.001 | 0.002 | -0.005 – 0.002 | 0.527 |
| Cognitively Unimpaired Aβ+ (72) | Zhang EN | 0.009 | 0.009 | -0.010 – 0.028 | 0.674 |
|  | Zhang BLUP | 0.006 | 0.010 | -0.014 – 0.026 | 0.674 |
|  | Hannum | -0.005 | 0.007 | -0.019 – 0.008 | 0.674 |
|  | Horvath | 0.000 | 0.007 | -0.014 – 0.013 | 0.951 |
|  | Phenoage | -0.005 | 0.005 | -0.016 – 0.005 | 0.674 |
| Cognitively Unimpaired Aβ- (167) | Zhang EN | 0.002 | 0.004 | -0.006 – 0.009 | 0.990 |
|  | Zhang BLUP | 0.003 | 0.004 | -0.005 – 0.010 | 0.990 |
|  | Hannum | -0.001 | 0.002 | -0.006 – 0.004 | 0.990 |
|  | Horvath | 0.000 | 0.002 | -0.005 – 0.004 | 0.990 |
|  | Phenoage | 0.000 | 0.002 | -0.004 – 0.004 | 0.990 |

Supplementary Table 6. AIBL longitudinal results for associations between accelerated ageing (DBAge) and PACC. *P* values shown represent values after FDR correction. Bolded values with ** represent values that remain significant after FDR correction. SE, standard error, CI 95, 95% confidence intervals, P Predictor, *p* value of clock used, EN Elastic Net, BLUP Best Linear Unbiased Prediction.

Supplementary Table 2. Cross-Sectional Amyloid-β (Aβ) Burden

| ­Population (n) | Predictor | Estimate | SE | CI 95 | *P* Predictor |
| --- | --- | --- | --- | --- | --- |
| Whole Cohort (373) | Zhang EN | -0.149 | 0.735 | -1.594 – 1.297 | 0.970 |
|  | Zhang BLUP | 0.027 | 0.710 | -1.369 – 1.422 | 0.970 |
|  | Hannum | 0.129 | 0.482 | -0.819 – 1.076 | 0.970 |
|  | Horvath | 0.086 | 0.425 | -0.750 – 0.922 | 0.970 |
|  | Phenoage | -0.166 | 0.360 | -0.874 – 0.541 | 0.970 |
| Aβ+ (171) | Zhang EN | 0.482 | 0.843 | -1.182 – 2.146 | 0.568 |
|  | Zhang BLUP | 0.898 | 0.804 | -0.691 – 2.486 | 0.333 |
|  | Hannum | 0.623 | 0.544 | -0.451 – 1.697 | 0.333 |
|  | Horvath | 0.597 | 0.456 | -0.303 – 1.498 | 0.333 |
|  | Phenoage | -0.566 | 0.412 | -1.380 – 0.248 | 0.333 |
| Aβ- (202) | Zhang EN | -0.131 | 0.237 | -0.599 – 0.336 | 0.709 |
|  | Zhang BLUP | -0.242 | 0.231 | -0.697 – 0.214 | 0.519 |
|  | Hannum | 0.175 | 0.159 | -0.138 – 0.488 | 0.519 |
|  | Horvath | 0.056 | 0.149 | -0.238 – 0.350 | 0.709 |
|  | Phenoage | -0.118 | 0.116 | -0.347 – 0.111 | 0.519 |
| Cognitively Unimpaired (240) | Zhang EN | -0.862 | 0.715 | -2.270 – 0.547 | 0.469 |
|  | Zhang BLUP | -1.365 | 0.693 | -2.73 – 0.001 | 0.250 |
|  | Hannum | -0.329 | 0.499 | -1.313 – 0.654 | 0.510 |
|  | Horvath | -0.501 | 0.464 | -1.415 – 0.413 | 0.469 |
|  | Phenoage | 0.305 | 0.371 | -0.426 – 1.035 | 0.510 |
| Cognitively Unimpaired Aβ+ (72) | Zhang EN | -1.185 | 1.204 | -3.592 – 1.222 | 0.696 |
|  | Zhang BLUP | -0.924 | 1.085 | -3.093 – 1.245 | 0.696 |
|  | Hannum | -0.296 | 0.904 | -2.103 – 1.511 | 0.786 |
|  | Horvath | -0.658 | 0.806 | -2.270 – 0.954 | 0.696 |
|  | Phenoage | -0.183 | 0.672 | -1.526 – 1.160 | 0.786 |
| Cognitively Unimpaired Aβ- (168) | Zhang EN | 0.064 | 0.264 | -0.457 – 0.585 | 0.809 |
|  | Zhang BLUP | -0.163 | 0.263 | -0.682 – 0.356 | 0.671 |
|  | Hannum | 0.293 | 0.180 | -0.064 – 0.649 | 0.533 |
|  | Horvath | 0.186 | 0.168 | -0.147 – 0.519 | 0.671 |
|  | Phenoage | -0.106 | 0.133 | -0.368 – 0.156 | 0.671 |

Supplementary Table 2. AIBL cross-sectional results for associations between accelerated ageing (DBAge) and Amyloid-β (Aβ) Burden. *P* values shown represent values after FDR correction. Bolded values with ** represent values that remain significant after FDR correction. SE, standard error, CI 95, 95% confidence intervals, P Predictor, *p* value of clock used, EN Elastic Net, BLUP Best Linear Unbiased Prediction.

Supplementary Table 7. AIBL Longitudinal Amyloid-β (Aβ) Burden

| ­Population (n) | Predictor | Estimate | SE | CI 95 | *P* Predictor |
| --- | --- | --- | --- | --- | --- |
| Whole Cohort (221) | Zhang EN | -0.051 | 0.083 | -0.214 – 0.111 | 0.666 |
|  | Zhang BLUP | -0.042 | 0.085 | -0.210 – 0.125 | 0.666 |
|  | Hannum | -0.025 | 0.057 | -0.137 – 0.088 | 0.666 |
|  | Horvath | -0.051 | 0.052 | -0.153 – 0.051 | 0.666 |
|  | Phenoage | -0.060 | 0.045 | -0.148 – 0.028 | 0.666 |
| Aβ+ (83) | Zhang EN | -0.142 | 0.148 | -0.437 – 0.153 | 0.342 |
|  | Zhang BLUP | -0.163 | 0.157 | -0.477 – 0.150 | 0.342 |
|  | Hannum | -0.102 | 0.107 | -0.315 – 0.110 | 0.342 |
|  | Horvath | -0.095 | 0.093 | -0.280 – 0.089 | 0.342 |
|  | Phenoage | -0.106 | 0.088 | -0.281 – 0.069 | 0.342 |
| Aβ- (138) | Zhang EN | 0.018 | 0.062 | -0.106 – 0.141 | 0.885 |
|  | Zhang BLUP | 0.009 | 0.064 | -0.116 – 0.135 | 0.885 |
|  | Hannum | 0.023 | 0.043 | -0.063 – 0.109 | 0.885 |
|  | Horvath | 0.011 | 0.040 | -0.069 – 0.091 | 0.885 |
|  | Phenoage | -0.023 | 0.033 | -0.088 – 0.041 | 0.885 |
| Cognitively Unimpaired (161) | Zhang EN | 0.065 | 0.080 | -0.092 – 0.223 | 0.855 |
|  | Zhang BLUP | 0.058 | 0.087 | -0.113 – 0.229 | 0.855 |
|  | Hannum | 0.017 | 0.056 | -0.094 – 0.128 | 0.947 |
|  | Horvath | -0.002 | 0.053 | -0.106 – 0.102 | 0.972 |
|  | Phenoage | -0.028 | 0.043 | -0.112 – 0.056 | 0.855 |
| Cognitively Unimpaired Aβ+ (42) | Zhang EN | 0.004 | 0.191 | -0.385 – 0.393 | 0.983 |
|  | Zhang BLUP | -0.006 | 0.203 | -0.420 – 0.408 | 0.983 |
|  | Hannum | -0.024 | 0.140 | -0.309 – 0.262 | 0.983 |
|  | Horvath | 0.057 | 0.142 | -0.232 – 0.345 | 0.983 |
|  | Phenoage | -0.045 | 0.120 | -0.289 – 0.199 | 0.983 |
| Cognitively Unimpaired Aβ- (119) | Zhang EN | 0.046 | 0.067 | -0.086 – 0.178 | 0.754 |
|  | Zhang BLUP | 0.037 | 0.072 | -0.105 – 0.180 | 0.754 |
|  | Hannum | 0.042 | 0.049 | -0.054 – 0.138 | 0.754 |
|  | Horvath | -0.012 | 0.043 | -0.098 – 0.074 | 0.780 |
|  | Phenoage | -0.023 | 0.034 | -0.091 – 0.045 | 0.754 |

Supplementary Table 7. AIBL longitudinal results for associations between accelerated ageing (DBAge) and Amyloid-β (Aβ) Burden. *P* values shown represent values after FDR correction. Bolded values with ** represent values that remain significant after FDR correction. SE, standard error, CI 95, 95% confidence intervals, P Predictor, *p* value of clock used, EN Elastic Net, BLUP Best Linear Unbiased Prediction.

Supplementary Table 10. AIBL Hippocampal Volume Longitudinal

| ­Population (n) | Predictor | Estimate | SE | CI 95 | *P* Predictor |
| --- | --- | --- | --- | --- | --- |
| Whole Cohort (186) | Zhang EN | -0.002 | 0.002 | -0.005 – 0.002 | 0.709 |
|  | Zhang BLUP | -0.002 | 0.002 | -0.005 – 0.002 | 0.709 |
|  | Hannum | -0.003 | 0.001 | -0.005 – 0.000 | 0.194 |
|  | Horvath | 0.000 | 0.001 | -0.002 – 0.002 | 0.974 |
|  | Phenoage | 0.001 | 0.001 | -0.001 – 0.002 | 0.741 |
| Aβ+ (77) | Zhang EN | -0.004 | 0.005 | -0.013 – 0.002 | 0.628 |
|  | Zhang BLUP | -0.002 | 0.004 | -0.011 – 0.006 | 0.628 |
|  | Hannum | -0.007 | 0.003 | -0.012 – -0.007 | 0.073 |
|  | Horvath | -0.002 | 0.003 | -0.008 – -0.001 | 0.628 |
|  | Phenoage | 0.002 | 0.002 | -0.003 – 0.004 | 0.628 |
| Aβ- (133) | Zhang EN | -0.001 | 0.002 | -0.005 – 0.006 | 0.975 |
|  | Zhang BLUP | -0.001 | 0.002 | -0.005 – 0.002 | 0.975 |
|  | Hannum | 0.000 | 0.001 | -0.003 – 0.002 | 0.975 |
|  | Horvath | 0.000 | 0.001 | -0.003 – 0.002 | 0.975 |
|  | Phenoage | 0.000 | 0.001 | -0.002 – 0.002 | 0.975 |
| Cognitively Unimpaired (165) | Zhang EN | 0.000 | 0.002 | -0.004 – 0.002 | 0.938 |
|  | Zhang BLUP | 0.000 | 0.002 | -0.003 – 0.003 | 0.938 |
|  | Hannum | -0.001 | 0.001 | -0.004 – 0.004 | 0.938 |
|  | Horvath | 0.000 | 0.001 | -0.002 – 0.001 | 0.938 |
|  | Phenoage | 0.000 | 0.001 | -0.002 – 0.002 | 0.938 |
| Cognitively Unimpaired Aβ+ (62) | Zhang EN | 0.001 | 0.004 | -0.007 – 0.001 | 0.935 |
|  | Zhang BLUP | 0.001 | 0.004 | -0.007 – 0.010 | 0.935 |
|  | Hannum | 0.000 | 0.003 | -0.006 – 0.006 | 0.935 |
|  | Horvath | 0.000 | 0.002 | -0.004 – 0.005 | 0.935 |
|  | Phenoage | 0.002 | 0.003 | -0.003 – 0.007 | 0.935 |
| Cognitively Unimpaired Aβ- (126) | Zhang EN | -0.001 | 0.002 | -0.005 – 0.003 | 0.891 |
|  | Zhang BLUP | -0.002 | 0.002 | -0.006 – 0.002 | 0.891 |
|  | Hannum | 0.000 | 0.001 | -0.003 – 0.003 | 0.891 |
|  | Horvath | 0.000 | 0.001 | -0.003 – 0.002 | 0.891 |
|  | Phenoage | 0.000 | 0.001 | -0.002 – 0.001 | 0.891 |

Supplementary Table 10. AIBL longitudinal results for associations between accelerated ageing (DBAge) and Hippocampal Volume. *P* values shown represent values after FDR correction. Bolded values with ** represent values that remain significant after FDR correction. SE, standard error, CI 95, 95% confidence intervals, P Predictor, *p* value of clock used, EN Elastic Net, BLUP Best Linear Unbiased Prediction.

Supplementary Table 11. AIBL Ventricles Longitudinal

| ­Population (n) | Predictor | Estimate | SE | CI 95 | *P* Predictor |
| --- | --- | --- | --- | --- | --- |
| Whole Cohort (186) | Zhang EN | 0.072 | 0.066 | -0.058 – 0.203 | 0.344 |
|  | Zhang BLUP | 0.060 | 0.065 | -0.069 – 0.189 | 0.359 |
|  | Hannum | 0.092 | 0.042 | 0.008 – 0.176 | 0.160 |
|  | Horvath | 0.054 | 0.041 | -0.027 – 0.135 | 0.316 |
|  | Phenoage | 0.044 | 0.032 | -0.020 – 0.108 | 0.316 |
| Aβ+ (77) | Zhang EN | 0.100 | 0.150 | -0.201 – 0.402 | 0.507 |
|  | Zhang BLUP | 0.132 | 0.148 | -0.164 – 0.428 | 0.507 |
|  | Hannum | 0.073 | 0.095 | -0.117 – 0.264 | 0.507 |
|  | Horvath | 0.064 | 0.095 | -0.126 – 0.254 | 0.507 |
|  | Phenoage | 0.064 | 0.075 | -0.086 – 0.214 | 0.507 |
| Aβ- (130) | Zhang EN | -0.001 | 0.071 | -0.141 – 0.140 | 0.991 |
|  | Zhang BLUP | 0.050 | 0.070 | -0.088 – 0.189 | 0.592 |
|  | Hannum | 0.053 | 0.046 | -0.039 – 0.144 | 0.434 |
|  | Horvath | 0.060 | 0.044 | -0.026 – 0.147 | 0.434 |
|  | Phenoage | 0.039 | 0.034 | -0.028 – 0.107 | 0.434 |
| Cognitively Unimpaired (165) | Zhang EN | 0.038 | 0.061 | -0.083 – 0.159 | 0.671 |
|  | Zhang BLUP | -0.005 | 0.060 | -0.124 – 0.114 | 0.933 |
|  | Hannum | 0.052 | 0.041 | -0.029 – 0.132 | 0.550 |
|  | Horvath | 0.028 | 0.037 | -0.044 – 0.101 | 0.671 |
|  | Phenoage | 0.038 | 0.031 | -0.023 – 0.098 | 0.550 |
| Cognitively Unimpaired Aβ+ (62) | Zhang EN | 0.020 | 0.130 | -0.243 – 0.284 | 0.977 |
|  | Zhang BLUP | 0.104 | 0.129 | -0.157 – 0.365 | 0.977 |
|  | Hannum | -0.003 | 0.095 | -0.195 – 0.190 | 0.977 |
|  | Horvath | -0.022 | 0.079 | -0.183 – 0.139 | 0.977 |
|  | Phenoage | -0.003 | 0.082 | -0.169 – 0.164 | 0.977 |
| Cognitively Unimpaired Aβ- (126) | Zhang EN | 0.000 | 0.069 | -0.137 – 0.137 | 0.999 |
|  | Zhang BLUP | 0.038 | 0.067 | -0.094 – 0.170 | 0.950 |
|  | Hannum | 0.009 | 0.046 | -0.083 – 0.100 | 0.999 |
|  | Horvath | 0.029 | 0.042 | -0.053 – 0.112 | 0.950 |
|  | Phenoage | 0.042 | 0.032 | -0.022 – 0.106 | 0.950 |

Supplementary Table 11. AIBL longitudinal results for associations between accelerated ageing (DBAge) and Ventricle Volume. *P* values shown represent values after FDR correction. Bolded values with ** represent values that remain significant after FDR correction. SE, standard error, CI 95, 95% confidence intervals, P Predictor, *p* value of clock used, EN Elastic Net, BLUP Best Linear Unbiased Prediction.

Supplementary Table 3. AIBL Cross-Sectional White Matter Volume

| ­Population (n) | Predictor | Estimate | SE | CI 95 | *P* Predictor |
| --- | --- | --- | --- | --- | --- |
| Whole Cohort (329) | Zhang EN | -0.544 | 0.479 | -1.487 – 0.398 | 0.486 |
|  | Zhang BLUP | -0.541 | 0.465 | -1.456 – 0.374 | 0.486 |
|  | Hannum | -0.008 | 0.334 | -0.666 – 0.649 | 0.981 |
|  | Horvath | -0.095 | 0.276 | -0.638 – 0.447 | 0.912 |
|  | Phenoage | -0.251 | 0.238 | -0.719 – 0.217 | 0.486 |
| Aβ+ (145) | Zhang EN | -0.350 | 0.742 | -1.818 – 1.117 | 0.904 |
|  | Zhang BLUP | 0.097 | 0.697 | -1.282 – 1.475 | 0.904 |
|  | Hannum | 0.059 | 0.487 | -0.905 – 1.022 | 0.904 |
|  | Horvath | 0.075 | 0.384 | -0.685 – 0.835 | 0.904 |
|  | Phenoage | -0.052 | 0.356 | -0.756 – 0.653 | 0.904 |
| Aβ- (184) | Zhang EN | -0.805 | 0.637 | -2.062 – 0.453 | 0.347 |
|  | Zhang BLUP | -1.198 | 0.637 | -2.455 – 0.059 | 0.216 |
|  | Hannum | -0.199 | 0.477 | -1.141 – 0.743 | 0.677 |
|  | Horvath | -0.398 | 0.420 | -1.226 – 0.431 | 0.431 |
|  | Phenoage | -0.566 | 0.328 | -1.214 – 0.082 | 0.216 |
| Cognitively Unimpaired (220) | Zhang EN | -0.924 | 0.569 | -2.046 – 0.198 | 0.265 |
|  | Zhang BLUP | -1.225 | 0.562 | -2.333 – -0.117 | 0.152 |
|  | Hannum | 0.200 | 0.429 | -0.645 – 1.046 | 0.641 |
|  | Horvath | -0.205 | 0.379 | -0.952 – 0.541 | 0.641 |
|  | Phenoage | -0.390 | 0.303 | -0.987 – 0.207 | 0.332 |
| Cognitively Unimpaired Aβ+ (65) | Zhang EN | -1.823 | 1.150 | -4.127 – 0.481 | 0.446 |
|  | Zhang BLUP | -1.441 | 1.057 | -3.560 – 0.678 | 0.446 |
|  | Hannum | 0.207 | 0.902 | -1.601 – 2.015 | 0.819 |
|  | Horvath | -0.542 | 0.788 | -2.120 – 1.037 | 0.618 |
|  | Phenoage | -0.563 | 0.719 | -2.003 – 0.878 | 0.618 |
| Cognitively Unimpaired Aβ- (155) | Zhang EN | -0.546 | 0.673 | -1.876 – 0.785 | 0.698 |
|  | Zhang BLUP | -1.028 | 0.683 | -2.378 – 0.322 | 0.515 |
|  | Hannum | 0.230 | 0.514 | -0.785 – 1.245 | 0.818 |
|  | Horvath | -0.039 | 0.444 | -0.917 – 0.838 | 0.929 |
|  | Phenoage | -0.433 | 0.341 | -1.108 – 0.241 | 0.515 |

Supplementary Table 3. AIBL cross-sectional results for associations between accelerated ageing (DBAge) and White Matter Volume. *P* values shown represent values after FDR correction. Bolded values with ** represent values that remain significant after FDR correction. SE, standard error, CI 95, 95% confidence intervals, P Predictor, *p* value of clock used, EN Elastic Net, BLUP Best Linear Unbiased Prediction.

Supplementary Table 4. AIBL Cross-Sectional Grey Matter Volume

| ­Population (n) | Predictor | Estimate | SE | CI 95 | *P* Predictor |
| --- | --- | --- | --- | --- | --- |
| Whole Cohort (329) | Zhang EN | 0.268 | 0.448 | -0.613 – 1.148 | 0.688 |
|  | Zhang BLUP | 0.620 | 0.434 | -0.233 – 1.473 | 0.579 |
|  | Hannum | -0.293 | 0.311 | -0.906 – 0.319 | 0.579 |
|  | Horvath | -0.034 | 0.257 | -0.540 – 0.472 | 0.896 |
|  | Phenoage | -0.246 | 0.222 | -0.683 – 0.190 | 0.579 |
| Aβ+ (145) | Zhang EN | -0.163 | 0.741 | -1.629 – 1.304 | 0.915 |
|  | Zhang BLUP | 0.423 | 0.695 | -0.952 – 1.798 | 0.906 |
|  | Hannum | -0.438 | 0.485 | -1.397 – 0.521 | 0.906 |
|  | Horvath | -0.041 | 0.384 | -0.800 – 0.718 | 0.915 |
|  | Phenoage | -0.254 | 0.355 | -0.957 – 0.448 | 0.906 |
| Aβ- (184) | Zhang EN | 0.401 | 0.494 | -0.575 – 1.376 | 0.751 |
|  | Zhang BLUP | 0.633 | 0.495 | -0.345 – 1.611 | 0.751 |
|  | Hannum | -0.189 | 0.369 | -0.918 – 0.541 | 0.751 |
|  | Horvath | -0.188 | 0.325 | -0.830 – 0.455 | 0.751 |
|  | Phenoage | -0.081 | 0.256 | -0.587 – 0.424 | 0.751 |
| Cognitively Unimpaired (220) | Zhang EN | 0.449 | 0.447 | -0.433 – 1.331 | 0.661 |
|  | Zhang BLUP | 0.705 | 0.442 | -0.167 – 1.577 | 0.561 |
|  | Hannum | -0.212 | 0.336 | -0.874 – 0.450 | 0.661 |
|  | Horvath | 0.073 | 0.297 | -0.512 – 0.658 | 0.805 |
|  | Phenoage | -0.191 | 0.238 | -0.660 – 0.277 | 0.661 |
| Cognitively Unimpaired Aβ+ (65) | Zhang EN | 0.549 | 0.974 | -1.402 – 2.501 | 0.876 |
|  | Zhang BLUP | 0.835 | 0.886 | -0.940 – 2.610 | 0.875 |
|  | Hannum | -0.119 | 0.750 | -1.621 – 1.383 | 0.876 |
|  | Horvath | 0.784 | 0.648 | -0.516 – 2.084 | 0.875 |
|  | Phenoage | 0.094 | 0.600 | -1.109 – 1.297 | 0.876 |
| Cognitively Unimpaired Aβ- (155) | Zhang EN | 0.314 | 0.520 | -0.713 – 1.342 | 0.546 |
|  | Zhang BLUP | 0.523 | 0.529 | -0.523 – 1.569 | 0.546 |
|  | Hannum | -0.329 | 0.396 | -1.111 – 0.453 | 0.546 |
|  | Horvath | -0.243 | 0.342 | -0.919 – 0.433 | 0.546 |
|  | Phenoage | -0.225 | 0.264 | -0.747 – 0.297 | 0.546 |

Supplementary Table 4. AIBL cross-sectional results for associations between accelerated ageing (DBAge) and Grey Matter Volume. *P* values shown represent values after FDR correction. Bolded values with ** represent values that remain significant after FDR correction. SE, standard error, CI 95, 95% confidence intervals, P Predictor, *p* value of clock used, EN Elastic Net, BLUP Best Linear Unbiased Prediction.

Supplementary Table 5. AIBL Cross-Sectional Ventricle Volume

| ­Population (n) | Predictor | Estimate | SE | CI 95 | *P* Predictor |
| --- | --- | --- | --- | --- | --- |
| Whole Cohort (329) | Zhang EN | 0.128 | 0.402 | -0.662 – 0.918 | 0.916 |
|  | Zhang BLUP | 0.041 | 0.390 | -0.726 – 0.808 | 0.916 |
|  | Hannum | 0.601 | 0.278 | 0.055 – 1.147 | 0.156 |
|  | Horvath | 0.301 | 0.230 | -0.152 – 0.753 | 0.320 |
|  | Phenoage | 0.274 | 0.199 | -0.117 – 0.664 | 0.320 |
| Aβ+ (145) | Zhang EN | 0.324 | 0.639 | -0.941 – 1.588 | 0.943 |
|  | Zhang BLUP | 0.158 | 0.600 | -1.029 – 1.346 | 0.943 |
|  | Hannum | 0.718 | 0.415 | -0.103 – 1.539 | 0.346 |
|  | Horvath | 0.489 | 0.328 | -0.160 – 1.139 | 0.346 |
|  | Phenoage | -0.022 | 0.307 | -0.629 – 0.585 | 0.943 |
| Aβ- (184) | Zhang EN | 0.036 | 0.513 | -0.977 – 1.048 | 0.948 |
|  | Zhang BLUP | -0.033 | 0.516 | -1.051 – 0.984 | 0.948 |
|  | Hannum | 0.595 | 0.380 | -0.156 – 1.346 | 0.299 |
|  | Horvath | 0.039 | 0.337 | -0.626 – 0.705 | 0.948 |
|  | Phenoage | 0.572 | 0.262 | 0.055 – 1.089 | 0.151 |
| Cognitively Unimpaired (220) | Zhang EN | 0.256 | 0.402 | -0.537 – 1.049 | 0.529 |
|  | Zhang BLUP | -0.266 | 0.399 | -1.053 – 0.521 | 0.529 |
|  | Hannum | 0.341 | 0.301 | -0.252 – 0.934 | 0.529 |
|  | Horvath | -0.168 | 0.266 | -0.693 – 0.357 | 0.529 |
|  | Phenoage | 0.268 | 0.213 | -0.152 – 0.688 | 0.529 |
| Cognitively Unimpaired Aβ+ (65) | Zhang EN | -0.199 | 0.835 | -1.872 – 1.473 | 0.812 |
|  | Zhang BLUP | -0.670 | 0.758 | -2.189 – 0.850 | 0.812 |
|  | Hannum | 0.187 | 0.641 | -1.097 – 1.471 | 0.812 |
|  | Horvath | -0.139 | 0.561 | -1.264 – 0.986 | 0.812 |
|  | Phenoage | -0.273 | 0.512 | -1.299 – 0.753 | 0.812 |
| Cognitively Unimpaired Aβ- (155) | Zhang EN | 0.461 | 0.480 | -0.487 – 1.410 | 0.563 |
|  | Zhang BLUP | -0.063 | 0.491 | -1.034 – 0.908 | 0.898 |
|  | Hannum | 0.455 | 0.365 | -0.267 – 1.176 | 0.537 |
|  | Horvath | -0.200 | 0.316 | -0.826 – 0.425 | 0.660 |
|  | Phenoage | 0.464 | 0.242 | -0.014 – 0.942 | 0.286 |

Supplementary Table 5. AIBL cross-sectional results for associations between accelerated ageing (DBAge) and Ventricle Volume. *P* values shown represent values after FDR correction. Bolded values with ** represent values that remain significant after FDR correction. SE, standard error, CI 95, 95% confidence intervals, P Predictor, *p* value of clock used, EN Elastic Net, BLUP Best Linear Unbiased Prediction.

Supplementary Table 8. AIBL Longitudinal White Matter Volume

| ­Population (n) | Predictor | Estimate | SE | CI 95 | *P* Predictor |
| --- | --- | --- | --- | --- | --- |
| Whole Cohort (186) | Zhang EN | 0.039 | 0.073 | -0.105 – 0.184 | 0.830 |
|  | Zhang BLUP | 0.045 | 0.071 | -0.096 – 0.186 | 0.830 |
|  | Hannum | 0.010 | 0.047 | -0.083 – 0.103 | 0.830 |
|  | Horvath | 0.045 | 0.046 | -0.046 – 0.135 | 0.830 |
|  | Phenoage | 0.009 | 0.036 | -0.063 – 0.080 | 0.830 |
| Aβ+ (77) | Zhang EN | 0.134 | 0.169 | -0.204 – 0.473 | 0.905 |
|  | Zhang BLUP | 0.077 | 0.165 | -0.253 – 0.407 | 0.905 |
|  | Hannum | 0.116 | 0.107 | -0.098 – 0.329 | 0.905 |
|  | Horvath | 0.017 | 0.107 | -0.197 – 0.231 | 0.905 |
|  | Phenoage | -0.010 | 0.085 | -0.180 – 0.160 | 0.905 |
| Aβ- (133) | Zhang EN | 0.056 | 0.081 | -0.104 – 0.215 | 0.926 |
|  | Zhang BLUP | 0.036 | 0.078 | -0.119 – 0.191 | 0.926 |
|  | Hannum | -0.007 | 0.053 | -0.111 – 0.097 | 0.926 |
|  | Horvath | 0.057 | 0.049 | -0.040 – 0.155 | 0.926 |
|  | Phenoage | -0.004 | 0.039 | -0.080 – 0.073 | 0.926 |
| Cognitively Unimpaired (165) | Zhang EN | -0.010 | 0.078 | -0.165 – 0.145 | 0.895 |
|  | Zhang BLUP | 0.048 | 0.077 | -0.104 – 0.200 | 0.752 |
|  | Hannum | 0.035 | 0.052 | -0.068 – 0.139 | 0.752 |
|  | Horvath | 0.025 | 0.047 | -0.068 – 0.117 | 0.752 |
|  | Phenoage | -0.025 | 0.039 | -0.103 – 0.052 | 0.752 |
| Cognitively Unimpaired Aβ+ (62) | Zhang EN | 0.135 | 0.233 | -0.339 – 0.608 | 0.879 |
|  | Zhang BLUP | -0.036 | 0.235 | -0.513 – 0.441 | 0.879 |
|  | Hannum | 0.218 | 0.167 | -0.121 – 0.558 | 0.879 |
|  | Horvath | -0.055 | 0.143 | -0.346 – 0.236 | 0.879 |
|  | Phenoage | -0.143 | 0.151 | -0.450 – 0.165 | 0.879 |
| Cognitively Unimpaired Aβ- (126) | Zhang EN | 0.045 | 0.081 | -0.116 – 0.206 | 0.726 |
|  | Zhang BLUP | 0.070 | 0.078 | -0.084 – 0.225 | 0.613 |
|  | Hannum | 0.070 | 0.054 | -0.037 – 0.177 | 0.488 |
|  | Horvath | 0.070 | 0.048 | -0.025 – 0.165 | 0.488 |
|  | Phenoage | -0.001 | 0.038 | -0.077 – 0.075 | 0.984 |

Supplementary Table 8. AIBL longitudinal results for associations between accelerated ageing (DBAge) and White Matter Volume. *P* values shown represent values after FDR correction. Bolded values with ** represent values that remain significant after FDR correction. SE, standard error, CI 95, 95% confidence intervals, P Predictor, *p* value of clock used, EN Elastic Net, BLUP Best Linear Unbiased Prediction.

Supplementary Table 9. AIBL Longitudinal Grey Matter Volume

| ­Population (n) | Predictor | Estimate | SE | CI 95 | *P* Predictor |
| --- | --- | --- | --- | --- | --- |
| Whole Cohort (186) | Zhang EN | -0.044 | 0.091 | -0.224 – 0.135 | 0.833 |
|  | Zhang BLUP | -0.034 | 0.089 | -0.210 – 0.141 | 0.833 |
|  | Hannum | -0.092 | 0.059 | -0.207 – 0.024 | 0.600 |
|  | Horvath | 0.012 | 0.057 | -0.101 – 0.125 | 0.833 |
|  | Phenoage | 0.026 | 0.045 | -0.063 – 0.115 | 0.833 |
| Aβ+ (77) | Zhang EN | -0.023 | 0.200 | -0.425 – 0.379 | 0.982 |
|  | Zhang BLUP | 0.053 | 0.197 | -0.342 – 0.447 | 0.982 |
|  | Hannum | -0.153 | 0.125 | -0.403 – 0.097 | 0.924 |
|  | Horvath | 0.003 | 0.127 | -0.252 – 0.258 | 0.982 |
|  | Phenoage | 0.092 | 0.101 | -0.111 – 0.295 | 0.924 |
| Aβ- (133) | Zhang EN | -0.096 | 0.097 | -0.287 – 0.096 | 0.722 |
|  | Zhang BLUP | -0.037 | 0.095 | -0.225 – 0.152 | 0.722 |
|  | Hannum | -0.076 | 0.063 | -0.201 – 0.049 | 0.722 |
|  | Horvath | -0.022 | 0.060 | -0.141 – 0.098 | 0.722 |
|  | Phenoage | -0.019 | 0.047 | -0.112 – 0.073 | 0.722 |
| Cognitively Unimpaired (165) | Zhang EN | -0.041 | 0.086 | -0.211 – 0.128 | 0.860 |
|  | Zhang BLUP | 0.036 | 0.084 | -0.131 – 0.203 | 0.860 |
|  | Hannum | -0.114 | 0.057 | -0.226 – -0.001 | 0.236 |
|  | Horvath | 0.009 | 0.052 | -0.093 – 0.111 | 0.860 |
|  | Phenoage | -0.013 | 0.043 | -0.098 – 0.073 | 0.860 |
| Cognitively Unimpaired Aβ+ (62) | Zhang EN | -0.055 | 0.210 | -0.481 – 0.372 | 0.797 |
|  | Zhang BLUP | 0.105 | 0.212 | -0.326 – 0.535 | 0.781 |
|  | Hannum | -0.247 | 0.148 | -0.549 – 0.054 | 0.522 |
|  | Horvath | 0.076 | 0.128 | -0.183 – 0.335 | 0.781 |
|  | Phenoage | 0.090 | 0.138 | -0.190 – 0.370 | 0.781 |
| Cognitively Unimpaired Aβ- (126) | Zhang EN | -0.055 | 0.210 | -0.481 – 0.372 | 0.797 |
|  | Zhang BLUP | 0.105 | 0.212 | -0.326 – 0.535 | 0.781 |
|  | Hannum | -0.247 | 0.148 | -0.549 – 0.054 | 0.522 |
|  | Horvath | 0.076 | 0.128 | -0.183 – 0.335 | 0.781 |
|  | Phenoage | 0.090 | 0.138 | -0.190 – 0.370 | 0.781 |

Supplementary Table 9. AIBL longitudinal results for associations between accelerated ageing (DBAge) and Grey Matter Volume. *P* values shown represent values after FDR correction. Bolded values with ** represent values that remain significant after FDR correction. SE, standard error, CI 95, 95% confidence intervals, P Predictor, *p* value of clock used, EN Elastic Net, BLUP Best Linear Unbiased Prediction.

Supplementary Table 12. AIBL Cross-Sectional Hippocampal Volume

| ­Population (n) | Predictor | Estimate | SE | CI 95 | *P* Predictor |
| --- | --- | --- | --- | --- | --- |
| Whole Cohort (358) | Zhang EN | -0.012 | 0.008 | -0.027 – 0.003 | 0.149 |
|  | Zhang BLUP | -0.029 | 0.009 | -0.047 – -0.011 | 0.007 |
|  | Hannum | -0.019 | 0.006 | -0.031 – -0.006 | 0.009 |
|  | Horvath | -0.021 | 0.013 | -0.047 – 0.005 | 0.149 |
|  | Phenoage | -0.009 | 0.013 | -0.034 – 0.016 | 0.471 |
| Aβ+ (159) | Zhang EN | -0.020 | 0.011 | -0.041 – 0.002 | 0.096 |
|  | Zhang BLUP | -0.045 | 0.014 | -0.072 – -0.018 | 0.003 |
|  | Hannum | -0.034 | 0.010 | -0.054 – -0.015 | 0.003 |
|  | Horvath | -0.050 | 0.021 | -0.092 – -0.008 | 0.032 |
|  | Phenoage | -0.030 | 0.020 | -0.070 – 0.100 | 0.135 |
| Aβ- (199) | Zhang EN | 0.002 | 0.010 | -0.017 – 0.022 | 0.875 |
|  | Zhang BLUP | -0.009 | 0.011 | -0.031 – 0.014 | 0.875 |
|  | Hannum | 0.002 | 0.008 | -0.014 – 0.017 | 0.875 |
|  | Horvath | 0.002 | 0.015 | -0.028 – 0.032 | 0.875 |
|  | Phenoage | 0.014 | 0.015 | -0.016 – 0.044 | 0.875 |
| Cognitively Unimpaired (239) | Zhang EN | -0.001 | 0.009 | -0.019 – 0.016 | 0.922 |
|  | Zhang BLUP | -0.024 | 0.010 | -0.043 – -0.004 | 0.099 |
|  | Hannum | -0.010 | 0.007 | -0.024 – 0.004 | 0.414 |
|  | Horvath | -0.014 | 0.014 | -0.041 – 0.013 | 0.507 |
|  | Phenoage | -0.001 | 0.014 | -0.028 – 0.025 | 0.922 |
| Cognitively Unimpaired Aβ+ (72) | Zhang EN | -0.013 | 0.018 | -0.049 – 0.023 | 0.484 |
|  | Zhang BLUP | -0.058 | 0.019 | -0.096 – -0.020 | 0.018 |
|  | Hannum | -0.038 | 0.016 | -0.069 – -0.007 | 0.030 |
|  | Horvath | -0.069 | 0.025 | -0.120 – -0.019 | 0.019 |
|  | Phenoage | -0.034 | 0.024 | -0.082 – 0.015 | 0.209 |
| Cognitively Unimpaired Aβ- (167) | Zhang EN | 0.004 | 0.016 | -0.029 – 0.036 | 0.828 |
|  | Zhang BLUP | 0.014 | 0.017 | -0.019 – 0.047 | 0.828 |
|  | Hannum | -0.015 | 0.012 | -0.039 – 0.010 | 0.828 |
|  | Horvath | 0.004 | 0.011 | -0.017 – 0.026 | 0.828 |
|  | Phenoage | -0.004 | 0.008 | -0.020 – 0.012 | 0.828 |

Supplementary Table 12. AIBL cross-sectional results for associations between accelerated ageing (DiffAge) and Hippocampal Volume. *P* values shown represent values after FDR correction. Bolded values with ** represent values that remain significant after FDR correction. SE, standard error, CI 95, 95% confidence intervals, P Predictor, *p* value of clock used, EN Elastic Net, BLUP Best Linear Unbiased Prediction.

Supplementary Table 13. AIBL Cross-Sectional PACC

| ­Population (n) | Predictor | Estimate | SE | CI 95 | *P* Predictor |
| --- | --- | --- | --- | --- | --- |
| Whole Cohort (358) | Zhang EN | 0.039 | 0.022 | -0.005 – 0.083 | 0.296 |
|  | Zhang BLUP | 0.026 | 0.022 | -0.017 – 0.068 | 0.392 |
|  | Hannum | 0.002 | 0.015 | -0.027 – 0.031 | 0.999 |
|  | Horvath | 0.020 | 0.013 | -0.005 – 0.046 | 0.296 |
|  | Phenoage | 0.000 | 0.011 | -0.022 – 0.022 | 0.999 |
| Aβ+ (159) | Zhang EN | 0.019 | 0.038 | -0.057 – 0.094 | 0.866 |
|  | Zhang BLUP | -0.001 | 0.036 | -0.073 – 0.070 | 0.970 |
|  | Hannum | -0.010 | 0.025 | -0.059 – 0.039 | 0.866 |
|  | Horvath | 0.011 | 0.021 | -0.030 – 0.052 | 0.866 |
|  | Phenoage | 0.010 | 0.019 | -0.027 – 0.048 | 0.866 |
| Aβ- (199) | Zhang EN | 0.041 | 0.021 | -0.001 – 0.083 | 0.171 |
|  | Zhang BLUP | 0.038 | 0.021 | -0.003 – 0.079 | 0.171 |
|  | Hannum | 0.007 | 0.014 | -0.021 – 0.035 | 0.775 |
|  | Horvath | 0.022 | 0.013 | -0.004 – 0.048 | 0.171 |
|  | Phenoage | 0.001 | 0.011 | -0.020 – 0.021 | 0.954 |
| Cognitively Unimpaired (239) | Zhang EN | 0.026 | 0.015 | -0.003 – 0.056 | 0.139 |
|  | Zhang BLUP | 0.028 | 0.015 | -0.001 – 0.056 | 0.139 |
|  | Hannum | 0.008 | 0.011 | -0.013 – 0.028 | 0.474 |
|  | Horvath | 0.019 | 0.010 | 0.000 – 0.038 | 0.139 |
|  | Phenoage | 0.007 | 0.008 | -0.008 – 0.023 | 0.422 |
| Cognitively Unimpaired Aβ+ (72) | Zhang EN | 0.038 | 0.030 | -0.022 – 0.098 | 0.344 |
|  | Zhang BLUP | 0.040 | 0.027 | -0.014 – 0.093 | 0.344 |
|  | Hannum | 0.022 | 0.022 | -0.023 – 0.066 | 0.422 |
|  | Horvath | 0.010 | 0.020 | -0.030 – 0.050 | 0.627 |
|  | Phenoage | 0.040 | 0.016 | 0.008 – 0.072 | 0.078 |
| Cognitively Unimpaired Aβ- (167) | Zhang EN | 0.022 | 0.018 | -0.013 – 0.057 | 0.463 |
|  | Zhang BLUP | 0.019 | 0.018 | -0.016 – 0.054 | 0.463 |
|  | Hannum | 0.005 | 0.012 | -0.020 – 0.029 | 0.780 |
|  | Horvath | 0.023 | 0.011 | 0.001 – 0.046 | 0.197 |
|  | Phenoage | 0.003 | 0.009 | -0.015 – 0.020 | 0.780 |

Supplementary Table 13. AIBL cross-sectional results for associations between accelerated ageing (DiffAge) and PACC. *P* values shown represent values after FDR correction. Bolded values with ** represent values that remain significant after FDR correction. SE, standard error, CI 95, 95% confidence intervals, P Predictor, *p* value of clock used, EN Elastic Net, BLUP Best Linear Unbiased Prediction.

Supplementary Table 14. Cross-Sectional Amyloid-β (Aβ) Burden

| ­Population (n) | Predictor | Estimate | SE | CI 95 | *P* Predictor |
| --- | --- | --- | --- | --- | --- |
| Whole Cohort (373) | Zhang EN | -0.150 | 0.735 | -1.596 – 1.296 | 0.971 |
|  | Zhang BLUP | 0.026 | 0.710 | -1.370 – 1.421 | 0.971 |
|  | Hannum | 0.128 | 0.482 | -0.820 – 1.075 | 0.971 |
|  | Horvath | 0.085 | 0.425 | -0.751 – 0.921 | 0.971 |
|  | Phenoage | -0.167 | 0.360 | -0.874 – 0.541 | 0.971 |
| Aβ+ (171) | Zhang EN | 0.477 | 0.843 | -1.187 – 2.142 | 0.572 |
|  | Zhang BLUP | 0.895 | 0.805 | -0.695 – 2.484 | 0.335 |
|  | Hannum | 0.620 | 0.544 | -0.454 – 1.694 | 0.335 |
|  | Horvath | 0.594 | 0.456 | -0.306 – 1.495 | 0.335 |
|  | Phenoage | -0.568 | 0.412 | -1.382 – 0.247 | 0.335 |
| Aβ- (202) | Zhang EN | -0.131 | 0.237 | -0.599 – 0.336 | 0.708 |
|  | Zhang BLUP | -0.241 | 0.231 | -0.697 – 0.214 | 0.519 |
|  | Hannum | 0.175 | 0.159 | -0.138 – 0.488 | 0.519 |
|  | Horvath | 0.056 | 0.149 | -0.238 – 0.350 | 0.708 |
|  | Phenoage | -0.118 | 0.116 | -0.346 – 0.111 | 0.519 |
| Cognitively Unimpaired (240) | Zhang EN | -0.861 | 0.715 | -2.270 – 0.548 | 0.470 |
|  | Zhang BLUP | -1.365 | 0.693 | -2.730 – 0.001 | 0.251 |
|  | Hannum | -0.329 | 0.499 | -1.313 – 0.655 | 0.510 |
|  | Horvath | -0.501 | 0.464 | -1.415 – 0.414 | 0.470 |
|  | Phenoage | 0.305 | 0.371 | -0.426 – 1.035 | 0.510 |
| Cognitively Unimpaired Aβ+ (72) | Zhang EN | -1.186 | 1.204 | -3.593 – 1.222 | 0.695 |
|  | Zhang BLUP | -0.924 | 1.085 | -3.093 – 1.245 | 0.695 |
|  | Hannum | -0.296 | 0.904 | -2.103 – 1.511 | 0.786 |
|  | Horvath | -0.658 | 0.806 | -2.270 – 0.953 | 0.695 |
|  | Phenoage | -0.183 | 0.672 | -1.526 – 1.160 | 0.786 |
| Cognitively Unimpaired Aβ- (168) | Zhang EN | 0.064 | 0.264 | -0.457 – 0.585 | 0.809 |
|  | Zhang BLUP | -0.163 | 0.263 | -0.682 – 0.357 | 0.671 |
|  | Hannum | 0.293 | 0.180 | -0.063 – 0.649 | 0.532 |
|  | Horvath | 0.186 | 0.168 | -0.146 – 0.519 | 0.671 |
|  | Phenoage | -0.106 | 0.133 | -0.368 – 0.157 | 0.671 |

Supplementary Table 14. AIBL cross-sectional results for associations between accelerated ageing (DiffAge) and Amyloid-β (Aβ) Burden. *P* values shown represent values after FDR correction. Bolded values with ** represent values that remain significant after FDR correction. SE, standard error, CI 95, 95% confidence intervals, P Predictor, *p* value of clock used, EN Elastic Net, BLUP Best Linear Unbiased Prediction.

Supplementary Table 15. AIBL Cross-Sectional White Matter Volume

| ­Population (n) | Predictor | Estimate | SE | CI 95 | *P* Predictor |
| --- | --- | --- | --- | --- | --- |
| Whole Cohort (329) | Zhang EN | -0.544 | 0.479 | -1.487 – 0.398 | 0.486 |
|  | Zhang BLUP | -0.541 | 0.465 | -1.457 – 0.374 | 0.486 |
|  | Hannum | -0.008 | 0.334 | -0.665 – 0.649 | 0.981 |
|  | Horvath | -0.095 | 0.276 | -0.638 – 0.447 | 0.912 |
|  | Phenoage | -0.251 | 0.238 | -0.719 – 0.217 | 0.486 |
| Aβ+ (145) | Zhang EN | -0.350 | 0.742 | -1.817 – 1.118 | 0.904 |
|  | Zhang BLUP | 0.097 | 0.697 | -1.282 – 1.476 | 0.904 |
|  | Hannum | 0.059 | 0.487 | -0.904 – 1.022 | 0.904 |
|  | Horvath | 0.075 | 0.384 | -0.685 – 0.835 | 0.904 |
|  | Phenoage | -0.052 | 0.356 | -0.756 – 0.653 | 0.904 |
| Aβ- (184) | Zhang EN | -0.804 | 0.637 | -2.061 – 0.453 | 0.347 |
|  | Zhang BLUP | -1.198 | 0.637 | -2.455 – 0.059 | 0.217 |
|  | Hannum | -0.199 | 0.477 | -1.141 – 0.743 | 0.677 |
|  | Horvath | -0.397 | 0.420 | -1.226 – 0.413 | 0.431 |
|  | Phenoage | -0.566 | 0.328 | -1.213 – 0.082 | 0.217 |
| Cognitively Unimpaired (220) | Zhang EN | -0.924 | 0.569 | -2.046 – 0.198 | 0.265 |
|  | Zhang BLUP | -1.224 | 0.562 | -2.332 – -0.116 | 0.153 |
|  | Hannum | 0.201 | 0.429 | -0.645 – 1.047 | 0.640 |
|  | Horvath | -0.205 | 0.379 | -0.951 – 0.542 | 0.640 |
|  | Phenoage | -0.390 | 0.303 | -0.987 – 0.207 | 0.332 |
| Cognitively Unimpaired Aβ+ (65) | Zhang EN | -1.822 | 1.150 | -4.127 – 0.482 | 0.447 |
|  | Zhang BLUP | -1.441 | 1.057 | -3.560 – 0.679 | 0.447 |
|  | Hannum | 0.208 | 0.902 | -1.600 – 2.017 | 0.818 |
|  | Horvath | -0.540 | 0.788 | -2.119 – 1.038 | 0.619 |
|  | Phenoage | -0.562 | 0.719 | -2.003 – 0.878 | 0.619 |
| Cognitively Unimpaired Aβ- (155) | Zhang EN | -0.545 | 0.673 | -1.876 – 0.785 | 0.699 |
|  | Zhang BLUP | -1.028 | 0.683 | -2.378 – 0.322 | 0.516 |
|  | Hannum | 0.231 | 0.513 | -0.784 – 1.246 | 0.817 |
|  | Horvath | -0.039 | 0.444 | -0.916 – 0.839 | 0.930 |
|  | Phenoage | -0.433 | 0.341 | -1.108 – 0.241 | 0.516 |

Supplementary Table 15. AIBL cross-sectional results for associations between accelerated ageing (DiffAge) and White Matter Volume. *P* values shown represent values after FDR correction. Bolded values with ** represent values that remain significant after FDR correction. SE, standard error, CI 95, 95% confidence intervals, P Predictor, *p* value of clock used, EN Elastic Net, BLUP Best Linear Unbiased Prediction.

Supplementary Table 16. AIBL Cross-Sectional Grey Matter Volume

| ­Population (n) | Predictor | Estimate | SE | CI 95 | *P* Predictor |
| --- | --- | --- | --- | --- | --- |
| Whole Cohort (329) | Zhang EN | 0.268 | 0.448 | -0.612 – 1.149 | 0.687 |
|  | Zhang BLUP | 0.621 | 0.434 | -0.232 – 1.474 | 0.580 |
|  | Hannum | -0.293 | 0.311 | -0.905 – 0.320 | 0.580 |
|  | Horvath | -0.033 | 0.257 | -0.539 – 0.473 | 0.897 |
|  | Phenoage | -0.246 | 0.222 | -0.683 – 0.190 | 0.580 |
| Aβ+ (145) | Zhang EN | -0.161 | 0.742 | -1.627 – 1.306 | 0.917 |
|  | Zhang BLUP | 0.425 | 0.695 | -0.951 – 1.800 | 0.904 |
|  | Hannum | -0.437 | 0.485 | -1.396 – 0.522 | 0.904 |
|  | Horvath | -0.040 | 0.384 | -0.799 – 0.719 | 0.917 |
|  | Phenoage | -0.254 | 0.355 | -0.956 – 0.449 | 0.904 |
| Aβ- (184) | Zhang EN | 0.400 | 0.494 | -0.576 – 1.376 | 0.751 |
|  | Zhang BLUP | 0.633 | 0.496 | -0.345 – 1.611 | 0.751 |
|  | Hannum | -0.189 | 0.369 | -0.918 – 0.540 | 0.751 |
|  | Horvath | -0.188 | 0.325 | -0.830 – 0.454 | 0.751 |
|  | Phenoage | -0.081 | 0.256 | -0.587 – 0.424 | 0.751 |
| Cognitively Unimpaired (220) | Zhang EN | 0.449 | 0.447 | -0.433 – 1.330 | 0.660 |
|  | Zhang BLUP | 0.705 | 0.442 | -0.167 – 1.577 | 0.561 |
|  | Hannum | -0.212 | 0.336 | -0.874 – 0.450 | 0.660 |
|  | Horvath | 0.073 | 0.297 | -0.512 – 0.658 | 0.806 |
|  | Phenoage | -0.191 | 0.238 | -0.660 – 0.277 | 0.660 |
| Cognitively Unimpaired Aβ+ (65) | Zhang EN | 0.550 | 0.974 | -1.402 – 2.502 | 0.875 |
|  | Zhang BLUP | 0.835 | 0.886 | -0.940 – 2.611 | 0.874 |
|  | Hannum | -0.118 | 0.750 | -1.621 – 1.384 | 0.875 |
|  | Horvath | 0.784 | 0.648 | -0.515 – 2.083 | 0.874 |
|  | Phenoage | 0.095 | 0.600 | -1.108 – 1.297 | 0.875 |
| Cognitively Unimpaired Aβ- (155) | Zhang EN | 0.314 | 0.520 | -0.714 – 1.341 | 0.547 |
|  | Zhang BLUP | 0.522 | 0.529 | -0.524 – 1.569 | 0.547 |
|  | Hannum | -0.330 | 0.396 | -1.111 – 0.452 | 0.547 |
|  | Horvath | -0.244 | 0.342 | -0.920 – 0.432 | 0.547 |
|  | Phenoage | -0.226 | 0.264 | -0.748 – 0.296 | 0.547 |

Supplementary Table 16. AIBL cross-sectional results for associations between accelerated ageing (DiffAge) and Grey Matter Volume. *P* values shown represent values after FDR correction. Bolded values with ** represent values that remain significant after FDR correction. SE, standard error, CI 95, 95% confidence intervals, P Predictor, *p* value of clock used, EN Elastic Net, BLUP Best Linear Unbiased Prediction.

Supplementary Table 17. AIBL Cross-Sectional Ventricle Volume

| ­Population (n) | Predictor | Estimate | SE | CI 95 | *P* Predictor |
| --- | --- | --- | --- | --- | --- |
| Whole Cohort (329) | Zhang EN | 0.127 | 0.402 | -0.663 – 0.917 | 0.917 |
|  | Zhang BLUP | 0.041 | 0.390 | -0.727 – 0.808 | 0.917 |
|  | Hannum | 0.600 | 0.278 | 0.054 – 1.146 | 0.156 |
|  | Horvath | 0.300 | 0.230 | -0.152 – 0.753 | 0.322 |
|  | Phenoage | 0.273 | 0.199 | -0.117 – 0.664 | 0.322 |
| Aβ+ (145) | Zhang EN | 0.322 | 0.639 | -0.942 – 1.586 | 0.942 |
|  | Zhang BLUP | 0.158 | 0.601 | -1.030 – 1.345 | 0.942 |
|  | Hannum | 0.717 | 0.415 | -0.104 – 1.538 | 0.348 |
|  | Horvath | 0.489 | 0.328 | -0.161 – 1.138 | 0.348 |
|  | Phenoage | -0.022 | 0.307 | -0.629 – 0.585 | 0.942 |
| Aβ- (184) | Zhang EN | 0.036 | 0.513 | -0.977 – 1.048 | 0.948 |
|  | Zhang BLUP | -0.034 | 0.516 | -1.051 – 0.984 | 0.948 |
|  | Hannum | 0.595 | 0.380 | -0.156 – 1.345 | 0.299 |
|  | Horvath | 0.039 | 0.337 | -0.626 – 0.705 | 0.948 |
|  | Phenoage | 0.572 | 0.262 | 0.055 – 1.089 | 0.151 |
| Cognitively Unimpaired (220) | Zhang EN | 0.256 | 0.402 | -0.537 – 1.049 | 0.529 |
|  | Zhang BLUP | -0.266 | 0.399 | -1.053 – 0.521 | 0.529 |
|  | Hannum | 0.341 | 0.301 | -0.252 – 0.934 | 0.529 |
|  | Horvath | -0.168 | 0.266 | -0.693 – 0.357 | 0.529 |
|  | Phenoage | 0.268 | 0.213 | -0.152 – 0.688 | 0.529 |
| Cognitively Unimpaired Aβ+ (65) | Zhang EN | -0.198 | 0.835 | -1.871 – 1.474 | 0.813 |
|  | Zhang BLUP | -0.669 | 0.758 | -2.188 – 0.850 | 0.813 |
|  | Hannum | 0.188 | 0.641 | -1.096 – 1.472 | 0.813 |
|  | Horvath | -0.138 | 0.561 | -1.263 – 0.987 | 0.813 |
|  | Phenoage | -0.273 | 0.512 | -1.299 – 0.753 | 0.813 |
| Cognitively Unimpaired Aβ- (155) | Zhang EN | 0.461 | 0.480 | -0.488 – 1.410 | 0.564 |
|  | Zhang BLUP | -0.063 | 0.491 | -1.035 – 0.908 | 0.897 |
|  | Hannum | 0.454 | 0.365 | -0.267 – 1.175 | 0.538 |
|  | Horvath | -0.200 | 0.316 | -0.826 – 0.425 | 0.660 |
|  | Phenoage | 0.464 | 0.242 | -0.014 – 0.942 | 0.286 |

Supplementary Table 17. AIBL cross-sectional results for associations between accelerated ageing (DiffAge) and Ventricle Volume. *P* values shown represent values after FDR correction. Bolded values with ** represent values that remain significant after FDR correction. SE, standard error, CI 95, 95% confidence intervals, P Predictor, *p* value of clock used, EN Elastic Net, BLUP Best Linear Unbiased Prediction.

Supplementary Table 18. AIBL Longitudinal PACC

| ­Population (n) | Predictor | Estimate | SE | CI 95 | *P* Predictor |
| --- | --- | --- | --- | --- | --- |
| Whole Cohort (358) | Zhang EN | -0.001 | 0.004 | -0.010 – 0.008 | 0.847 |
|  | Zhang BLUP | -0.003 | 0.005 | -0.012 – 0.006 | 0.693 |
|  | Hannum | -0.005 | 0.003 | -0.011 – 0.001 | 0.644 |
|  | Horvath | -0.002 | 0.003 | -0.007 – 0.004 | 0.693 |
|  | Phenoage | -0.002 | 0.002 | -0.006 – 0.003 | 0.693 |
| Aβ+ (159) | Zhang EN | -0.004 | 0.011 | -0.026 – 0.019 | 0.913 |
|  | Zhang BLUP | -0.018 | 0.011 | -0.040 – 0.004 | 0.282 |
|  | Hannum | -0.012 | 0.008 | -0.028 – 0.003 | 0.282 |
|  | Horvath | -0.003 | 0.008 | -0.018 – 0.013 | 0.913 |
|  | Phenoage | -0.001 | 0.006 | -0.013 – 0.012 | 0.913 |
| Aβ- (199) | Zhang EN | 0.001 | 0.004 | -0.006 – 0.008 | 0.916 |
|  | Zhang BLUP | 0.005 | 0.004 | -0.002 – 0.012 | 0.825 |
|  | Hannum | -0.001 | 0.002 | -0.006 – 0.003 | 0.916 |
|  | Horvath | 0.000 | 0.002 | -0.004 – 0.005 | 0.916 |
|  | Phenoage | 0.000 | 0.002 | -0.004 – 0.003 | 0.916 |
| Cognitively Unimpaired (239) | Zhang EN | 0.005 | 0.004 | -0.001 – 0.012 | 0.300 |
|  | Zhang BLUP | 0.006 | 0.004 | -0.001 – 0.013 | 0.300 |
|  | Hannum | -0.001 | 0.002 | -0.006 – 0.003 | 0.687 |
|  | Horvath | 0.001 | 0.002 | -0.003 – 0.005 | 0.687 |
|  | Phenoage | -0.001 | 0.002 | -0.005 – 0.003 | 0.687 |
| Cognitively Unimpaired Aβ+ (72) | Zhang EN | 0.012 | 0.009 | -0.006 – 0.30 | 0.729 |
|  | Zhang BLUP | 0.009 | 0.010 | -0.011 – 0.028 | 0.729 |
|  | Hannum | -0.002 | 0.007 | -0.016 – 0.011 | 0.729 |
|  | Horvath | 0.003 | 0.006 | -0.010 – 0.016 | 0.729 |
|  | Phenoage | -0.004 | 0.005 | -0.015 – 0.006 | 0.729 |
| Cognitively Unimpaired Aβ- (167) | Zhang EN | 0.002 | 0.004 | -0.005 – 0.009 | 0.914 |
|  | Zhang BLUP | 0.003 | 0.004 | -0.004 – 0.010 | 0.914 |
|  | Hannum | -0.001 | 0.002 | -0.005 – 0.004 | 0.914 |
|  | Horvath | 0.000 | 0.002 | -0.004 – 0.005 | 0.914 |
|  | Phenoage | 0.000 | 0.002 | -0.003 – 0.004 | 0.914 |

Supplementary Table 18. AIBL longitudinal results for associations between accelerated ageing (DiffAge) and PACC. *P* values shown represent values after FDR correction. Bolded values with ** represent values that remain significant after FDR correction. SE, standard error, CI 95, 95% confidence intervals, P Predictor, *p* value of clock used, EN Elastic Net, BLUP Best Linear Unbiased Prediction.

Supplementary Table 19. AIBL Longitudinal Amyloid-β (Aβ) Burden

| ­Population (n) | Predictor | Estimate | SE | CI 95 | *P* Predictor |
| --- | --- | --- | --- | --- | --- |
| Whole Cohort (221) | Zhang EN | -0.051 | 0.083 | -0.214 – 0.112 | 0.668 |
|  | Zhang BLUP | -0.042 | 0.085 | -0.210 – 0.126 | 0.668 |
|  | Hannum | -0.025 | 0.057 | -0.137 – 0.088 | 0.668 |
|  | Horvath | -0.051 | 0.052 | -0.153 – 0.051 | 0.668 |
|  | Phenoage | -0.060 | 0.045 | -0.148 – 0.028 | 0.668 |
| Aβ+ (83) | Zhang EN | -0.141 | 0.148 | -0.436 – 0.154 | 0.343 |
|  | Zhang BLUP | -0.163 | 0.157 | -0.477 – 0.150 | 0.343 |
|  | Hannum | -0.102 | 0.107 | -0.314 – 0.110 | 0.343 |
|  | Horvath | -0.095 | 0.093 | -0.280 – 0.090 | 0.343 |
|  | Phenoage | -0.106 | 0.088 | -0.281 – 0.069 | 0.343 |
| Aβ- (138) | Zhang EN | 0.018 | 0.062 | -0.106 – 0.141 | 0.885 |
|  | Zhang BLUP | 0.009 | 0.064 | -0.116 – 0.135 | 0.885 |
|  | Hannum | 0.023 | 0.043 | -0.063 – 0.109 | 0.885 |
|  | Horvath | 0.011 | 0.040 | -0.069 – 0.091 | 0.885 |
|  | Phenoage | -0.023 | 0.033 | -0.088 – 0.041 | 0.885 |
| Cognitively Unimpaired (161) | Zhang EN | 0.066 | 0.080 | -0.092 – 0.223 | 0.855 |
|  | Zhang BLUP | 0.058 | 0.087 | -0.113 – 0.229 | 0.855 |
|  | Hannum | 0.017 | 0.056 | -0.094 – 0.128 | 0.946 |
|  | Horvath | -0.002 | 0.053 | -0.106 – 0.102 | 0.973 |
|  | Phenoage | -0.028 | 0.043 | -0.112 – 0.056 | 0.855 |
| Cognitively Unimpaired Aβ+ (42) | Zhang EN | 0.004 | 0.191 | -0.385 – 0.393 | 0.983 |
|  | Zhang BLUP | -0.006 | 0.203 | -0.420 – 0.408 | 0.983 |
|  | Hannum | -0.024 | 0.140 | -0.309 – 0.262 | 0.983 |
|  | Horvath | 0.057 | 0.142 | -0.231 – 0.345 | 0.983 |
|  | Phenoage | -0.045 | 0.120 | -0.289 – 0.199 | 0.983 |
| Cognitively Unimpaired Aβ- (119) | Zhang EN | 0.046 | 0.067 | -0.086 – 0.178 | 0.754 |
|  | Zhang BLUP | 0.037 | 0.072 | -0.105 – 0.180 | 0.754 |
|  | Hannum | 0.042 | 0.049 | -0.054 – 0.138 | 0.754 |
|  | Horvath | -0.012 | 0.043 | -0.098 – 0.074 | 0.780 |
|  | Phenoage | -0.023 | 0.034 | -0.091 – 0.045 | 0.754 |

Supplementary Table 19. AIBL longitudinal results for associations between accelerated ageing (DiffAge) and Amyloid-β (Aβ) Burden. *P* values shown represent values after FDR correction. Bolded values with ** represent values that remain significant after FDR correction. SE, standard error, CI 95, 95% confidence intervals, P Predictor, *p* value of clock used, EN Elastic Net, BLUP Best Linear Unbiased Prediction.

Supplementary Table 20. AIBL Longitudinal White Matter Volume

| ­Population (n) | Predictor | Estimate | SE | CI 95 | *P* Predictor |
| --- | --- | --- | --- | --- | --- |
| Whole Cohort (186) | Zhang EN | 0.062 | 0.071 | -0.079 – 0.203 | 0.650 |
|  | Zhang BLUP | 0.062 | 0.071 | -0.078 – 0.201 | 0.650 |
|  | Hannum | 0.025 | 0.047 | -0.067 – 0.117 | 0.681 |
|  | Horvath | 0.059 | 0.044 | -0.027 – 0.146 | 0.650 |
|  | Phenoage | 0.015 | 0.035 | -0.055 – 0.084 | 0.681 |
| Aβ+ (77) | Zhang EN | 0.158 | 0.166 | -0.175 – 0.491 | 0.866 |
|  | Zhang BLUP | 0.095 | 0.164 | -0.233 – 0.423 | 0.907 |
|  | Hannum | 0.138 | 0.109 | -0.080 – 0.356 | 0.866 |
|  | Horvath | 0.037 | 0.105 | -0.173 – 0.247 | 0.907 |
|  | Phenoage | -0.003 | 0.084 | -0.171 – 0.165 | 0.972 |
| Aβ- (133) | Zhang EN | 0.078 | 0.079 | -0.078 – 0.234 | 0.805 |
|  | Zhang BLUP | 0.053 | 0.077 | -0.100 – 0.206 | 0.829 |
|  | Hannum | 0.009 | 0.051 | -0.093 – 0.110 | 0.947 |
|  | Horvath | 0.071 | 0.047 | -0.022 – 0.164 | 0.676 |
|  | Phenoage | 0.003 | 0.038 | -0.072 – 0.077 | 0.947 |
| Cognitively Unimpaired (165) | Zhang EN | 0.008 | 0.076 | -0.142 – 0.158 | 0.917 |
|  | Zhang BLUP | 0.059 | 0.076 | -0.090 – 0.209 | 0.741 |
|  | Hannum | 0.045 | 0.051 | -0.056 – 0.145 | 0.741 |
|  | Horvath | 0.034 | 0.045 | -0.054 – 0.123 | 0.741 |
|  | Phenoage | -0.019 | 0.038 | -0.095 – 0.056 | 0.770 |
| Cognitively Unimpaired Aβ+ (62) | Zhang EN | 0.012 | 0.009 | -0.006 – 0.030 | 0.729 |
|  | Zhang BLUP | 0.009 | 0.010 | -0.011 – 0.028 | 0.729 |
|  | Hannum | -0.002 | 0.007 | -0.016 – 0.011 | 0.729 |
|  | Horvath | 0.003 | 0.006 | -0.010 – 0.016 | 0.729 |
|  | Phenoage | -0.004 | 0.005 | -0.015 – 0.006 | 0.729 |
| Cognitively Unimpaired Aβ- (126) | Zhang EN | 0.063 | 0.080 | -0.095 – 0.221 | 0.540 |
|  | Zhang BLUP | 0.082 | 0.077 | -0.070 – 0.234 | 0.480 |
|  | Hannum | 0.077 | 0.052 | -0.026 – 0.179 | 0.350 |
|  | Horvath | 0.077 | 0.046 | -0.013 – 0.168 | 0.350 |
|  | Phenoage | 0.004 | 0.037 | -0.070 – 0.077 | 0.922 |

Supplementary Table 20. AIBL longitudinal results for associations between accelerated ageing (DiffAge) and White Matter Volume. *P* values shown represent values after FDR correction. Bolded values with ** represent values that remain significant after FDR correction. SE, standard error, CI 95, 95% confidence intervals, P Predictor, *p* value of clock used, EN Elastic Net, BLUP Best Linear Unbiased Prediction.

Supplementary Table 21. AIBL Longitudinal Grey Matter Volume

| ­Population (n) | Predictor | Estimate | SE | CI 95 | *P* Predictor |
| --- | --- | --- | --- | --- | --- |
| Whole Cohort (186) | Zhang EN | -0.006 | 0.089 | -0.182 – 0.170 | 0.946 |
|  | Zhang BLUP | -0.008 | 0.088 | -0.182 – 0.166 | 0.946 |
|  | Hannum | -0.067 | 0.058 | -0.181 – 0.048 | 0.811 |
|  | Horvath | 0.039 | 0.055 | -0.071 – 0.148 | 0.811 |
|  | Phenoage | 0.034 | 0.044 | -0.053 – 0.121 | 0.811 |
| Aβ+ (77) | Zhang EN | 0.017 | 0.198 | -0.380 – 0.415 | 0.931 |
|  | Zhang BLUP | 0.080 | 0.195 | -0.312 – 0.472 | 0.931 |
|  | Hannum | -0.135 | 0.128 | -0.391 – 0.121 | 0.802 |
|  | Horvath | 0.033 | 0.124 | -0.216 – 0.282 | 0.931 |
|  | Phenoage | 0.100 | 0.100 | -0.100 – 0.300 | 0.802 |
| Aβ- (133) | Zhang EN | -0.061 | 0.095 | -0.250 – 0.127 | 0.967 |
|  | Zhang BLUP | -0.014 | 0.094 | -0.200 – 0.173 | 0.967 |
|  | Hannum | -0.053 | 0.062 | -0.176 – 0.069 | 0.967 |
|  | Horvath | 0.002 | 0.058 | -0.113 – 0.117 | 0.967 |
|  | Phenoage | -0.011 | 0.046 | -0.101 – 0.079 | 0.967 |
| Cognitively Unimpaired (165) | Zhang EN | -0.011 | 0.083 | -0.175 – 0.153 | 0.914 |
|  | Zhang BLUP | 0.055 | 0.083 | -0.109 – 0.219 | 0.914 |
|  | Hannum | -0.088 | 0.055 | -0.198 – 0.021 | 0.559 |
|  | Horvath | 0.027 | 0.049 | -0.070 – 0.125 | 0.914 |
|  | Phenoage | -0.005 | 0.042 | -0.087 – 0.078 | 0.914 |
| Cognitively Unimpaired Aβ+ (62) | Zhang EN | -0.013 | 0.203 | -0.424 – 0.399 | 0.950 |
|  | Zhang BLUP | 0.129 | 0.207 | -0.291 – 0.549 | 0.670 |
|  | Hannum | -0.215 | 0.150 | -0.519 – 0.089 | 0.670 |
|  | Horvath | 0.097 | 0.122 | -0.150 – 0.345 | 0.670 |
|  | Phenoage | 0.101 | 0.133 | -0.169 – 0.371 | 0.670 |
| Cognitively Unimpaired Aβ- (126) | Zhang EN | -0.071 | 0.095 | -0.260 – 0.118 | 0.763 |
|  | Zhang BLUP | -0.010 | 0.093 | -0.195 – 0.176 | 0.918 |
|  | Hannum | -0.070 | 0.062 | -0.194 – 0.053 | 0.763 |
|  | Horvath | -0.020 | 0.056 | -0.132 – 0.091 | 0.898 |
|  | Phenoage | -0.038 | 0.045 | -0.126 – 0.051 | 0.763 |

Supplementary Table 21. AIBL longitudinal results for associations between accelerated ageing (DiffAge) and Grey Matter Volume. *P* values shown represent values after FDR correction. Bolded values with ** represent values that remain significant after FDR correction. SE, standard error, CI 95, 95% confidence intervals, P Predictor, *p* value of clock used, EN Elastic Net, BLUP Best Linear Unbiased Prediction.

Supplementary Table 22. AIBL Hippocampal Volume Longitudinal

| ­Population (n) | Predictor | Estimate | SE | CI 95 | *P* Predictor |
| --- | --- | --- | --- | --- | --- |
| Whole Cohort (186) | Zhang EN | 0.000 | 0.002 | -0.004 – 0.003 | 0.878 |
|  | Zhang BLUP | -0.001 | 0.002 | -0.005 – 0.003 | 0.848 |
|  | Hannum | -0.002 | 0.001 | -0.004 – 0.001 | 0.757 |
|  | Horvath | 0.001 | 0.001 | -0.001 – 0.003 | 0.790 |
|  | Phenoage | 0.001 | 0.001 | -0.001 – 0.003 | 0.790 |
| Aβ+ (77) | Zhang EN | -0.002 | 0.004 | -0.011 – 0.007 | 0.800 |
|  | Zhang BLUP | -0.001 | 0.004 | -0.010 – 0.008 | 0.800 |
|  | Hannum | -0.006 | 0.003 | -0.012 – -0.001 | 0.157 |
|  | Horvath | -0.001 | 0.003 | -0.007 – 0.005 | 0.800 |
|  | Phenoage | 0.002 | 0.002 | -0.003 – 0.007 | 0.800 |
| Aβ- (133) | Zhang EN | 0.000 | 0.002 | -0.004 – 0.003 | 0.848 |
|  | Zhang BLUP | -0.001 | 0.002 | -0.004 – 0.003 | 0.848 |
|  | Hannum | 0.000 | 0.001 | -0.002 – 0.003 | 0.848 |
|  | Horvath | 0.000 | 0.001 | -0.002 – 0.003 | 0.848 |
|  | Phenoage | 0.000 | 0.001 | -0.002 – 0.002 | 0.848 |
| Cognitively Unimpaired (165) | Zhang EN | 0.001 | 0.002 | -0.003 – 0.004 | 0.879 |
|  | Zhang BLUP | 0.001 | 0.002 | -0.002 – 0.004 | 0.879 |
|  | Hannum | -0.001 | 0.001 | -0.003 – 0.002 | 0.879 |
|  | Horvath | 0.001 | 0.001 | -0.001 – 0.003 | 0.879 |
|  | Phenoage | 0.000 | 0.001 | -0.002 – 0.002 | 0.879 |
| Cognitively Unimpaired Aβ+ (62) | Zhang EN | 0.002 | 0.004 | -0.006 – 0.010 | 0.926 |
|  | Zhang BLUP | 0.001 | 0.004 | -0.007 – 0.009 | 0.926 |
|  | Hannum | 0.000 | 0.003 | -0.006 – 0.006 | 0.926 |
|  | Horvath | 0.001 | 0.002 | -0.004 – 0.005 | 0.926 |
|  | Phenoage | 0.002 | 0.002 | -0.003 – 0.007 | 0.926 |
| Cognitively Unimpaired Aβ- (126) | Zhang EN | 0.000 | 0.002 | -0.004 – 0.004 | 0.904 |
|  | Zhang BLUP | -0.001 | 0.002 | -0.005 – 0.003 | 0.904 |
|  | Hannum | 0.000 | 0.001 | -0.002 – 0.003 | 0.904 |
|  | Horvath | 0.000 | 0.001 | -0.002 – 0.003 | 0.904 |
|  | Phenoage | 0.000 | 0.001 | -0.002 – 0.002 | 0.904 |

Supplementary Table 22. AIBL longitudinal results for associations between accelerated ageing (DiffAge) and Hippocampal Volume. *P* values shown represent values after FDR correction. Bolded values with ** represent values that remain significant after FDR correction. SE, standard error, CI 95, 95% confidence intervals, P Predictor, *p* value of clock used, EN Elastic Net, BLUP Best Linear Unbiased Prediction.

Supplementary Table 23. AIBL Ventricles Longitudinal

| ­Population (n) | Predictor | Estimate | SE | CI 95 | *P* Predictor |
| --- | --- | --- | --- | --- | --- |
| Whole Cohort (186) | Zhang EN | 0.055 | 0.064 | -0.072 – 0.183 | 0.451 |
|  | Zhang BLUP | 0.049 | 0.065 | -0.079 – 0.177 | 0.451 |
|  | Hannum | 0.081 | 0.042 | -0.002 – 0.163 | 0.280 |
|  | Horvath | 0.040 | 0.040 | -0.038 – 0.119 | 0.451 |
|  | Phenoage | 0.038 | 0.032 | -0.024 – 0.101 | 0.451 |
| Aβ+ (77) | Zhang EN | 0.087 | 0.148 | -0.209 – 0.384 | 0.566 |
|  | Zhang BLUP | 0.123 | 0.147 | -0.171 – 0.417 | 0.566 |
|  | Hannum | 0.070 | 0.097 | -0.124 – 0.264 | 0.566 |
|  | Horvath | 0.053 | 0.092 | -0.132 – 0.239 | 0.566 |
|  | Phenoage | 0.060 | 0.074 | -0.088 – 0.208 | 0.566 |
| Aβ- (130) | Zhang EN | -0.024 | 0.070 | -0.162 – 0.114 | 0.729 |
|  | Zhang BLUP | 0.032 | 0.069 | -0.105 – 0.169 | 0.729 |
|  | Hannum | 0.035 | 0.045 | -0.054 – 0.125 | 0.729 |
|  | Horvath | 0.039 | 0.042 | -0.044 – 0.123 | 0.729 |
|  | Phenoage | 0.032 | 0.033 | -0.034 – 0.098 | 0.729 |
| Cognitively Unimpaired (165) | Zhang EN | 0.032 | 0.059 | -0.085 – 0.149 | 0.733 |
|  | Zhang BLUP | -0.007 | 0.059 | -0.124 – 0.110 | 0.904 |
|  | Hannum | 0.046 | 0.039 | -0.032 – 0.124 | 0.611 |
|  | Horvath | 0.024 | 0.035 | -0.046 – 0.093 | 0.733 |
|  | Phenoage | 0.035 | 0.030 | -0.024 – 0.093 | 0.611 |
| Cognitively Unimpaired Aβ+ (62) | Zhang EN | 0.021 | 0.125 | -0.233 – 0.275 | 0.994 |
|  | Zhang BLUP | 0.101 | 0.126 | -0.154 – 0.357 | 0.994 |
|  | Hannum | -0.001 | 0.095 | -0.193 – 0.192 | 0.994 |
|  | Horvath | -0.019 | 0.076 | -0.172 – 0.135 | 0.994 |
|  | Phenoage | -0.001 | 0.080 | -0.163 – 0.161 | 0.994 |
| Cognitively Unimpaired Aβ- (126) | Zhang EN | -0.018 | 0.068 | -0.152 – 0.117 | 0.946 |
|  | Zhang BLUP | 0.025 | 0.066 | -0.106 – 0.155 | 0.946 |
|  | Hannum | -0.003 | 0.044 | -0.091 – 0.085 | 0.946 |
|  | Horvath | 0.015 | 0.040 | -0.064 – 0.094 | 0.946 |
|  | Phenoage | 0.036 | 0.031 | -0.026 – 0.098 | 0.946 |

Supplementary Table 23. AIBL longitudinal results for associations between accelerated ageing (DiffAge) and Ventricle Volume. *P* values shown represent values after FDR correction. Bolded values with ** represent values that remain significant after FDR correction. SE, standard error, CI 95, 95% confidence intervals, P Predictor, *p* value of clock used, EN Elastic Net, BLUP Best Linear Unbiased Prediction.

Supplementary Table 24. ADNI Cross-Sectional Hippocampal Volume

| ­Population (n) | Predictor | Estimate | SE | CI 95 | *P* Predictor |
| --- | --- | --- | --- | --- | --- |
| Whole Cohort (382) | Zhang EN | -0.004 | 0.006 | -0.016 – 0.007 | 0.482 |
|  | Zhang BLUP | -0.004 | 0.006 | -0.015 – 0.007 | 0.445 |
|  | Hannum | -0.002 | 0.004 | -0.011 – 0.006 | 0.602 |
|  | Horvath | -0.003 | 0.004 | -0.010 – 0.004 | 0.425 |
|  | Phenoage | -0.003 | 0.003 | -0.010 – 0.003 | 0.344 |
| Aβ+ (194) | Zhang EN | -0.007 | 0.009 | -0.025 – 0.010 | 0.392 |
|  | Zhang BLUP | -0.006 | 0.008 | -0.022 – 0.010 | 0.428 |
|  | Hannum | -0.005 | 0.006 | -0.017 – 0.006 | 0.371 |
|  | Horvath | -0.006 | 0.005 | -0.016 – 0.003 | 0.193 |
|  | Phenoage | -0.007 | 0.005 | -0.016 – 0.002 | 0.146 |
| Aβ- (188) | Zhang EN | -0.001 | 0.008 | -0.017 – 0.015 | 0.866 |
|  | Zhang BLUP | -0.001 | 0.008 | -0.016 – 0.014 | 0.862 |
|  | Hannum | 0.005 | 0.006 | -0.008 – 0.017 | 0.471 |
|  | Horvath | 0.000 | 0.005 | -0.010 – 0.011 | 0.955 |
|  | Phenoage | 0.002 | 0.005 | -0.008 – 0.011 | 0.727 |
| Cognitively Unimpaired (117) | Zhang EN | -0.004 | 0.008 | -0.019 – 0.011 | 0.568 |
|  | Zhang BLUP | -0.008 | 0.007 | -0.021 – 0.006 | 0.264 |
|  | Hannum | -0.013 | 0.006 | -0.025 – 0.000 | 0.043 |
|  | Horvath | 0.000 | 0.005 | -0.009 – 0.010 | 0.920 |
|  | Phenoage | -0.002 | 0.004 | -0.011 – 0.007 | 0.662 |
| Cognitively Unimpaired Aβ+ (34) | Zhang EN | -0.033 | 0.020 | -0.074 – 0.008 | 0.111 |
|  | Zhang BLUP | -0.031 | 0.016 | -0.064 – 0.002 | 0.061 |
|  | Hannum | -0.029 | 0.014 | -0.057 – -0.001 | 0.046 |
|  | Horvath | 0.005 | 0.011 | -0.018 – 0.029 | 0.647 |
|  | Phenoage | -0.013 | 0.011 | -0.036 – 0.011 | 0.268 |
| Cognitively Unimpaired Aβ- (83) | Zhang EN | -0.002 | 0.009 | -0.019 – 0.016 | 0.825 |
|  | Zhang BLUP | -0.002 | 0.008 | -0.018 – 0.013 | 0.770 |
|  | Hannum | -0.011 | 0.008 | -0.027 – 0.004 | 0.143 |
|  | Horvath | -0.004 | 0.006 | -0.015 – 0.008 | 0.544 |
|  | Phenoage | -0.001 | 0.005 | -0.012 – 0.009 | 0.802 |

Supplementary Table 24. ADNI cross-sectional validation results for associations between accelerated ageing (DiffAge) hippocampal volume. *P* values shown represent values before FDR correction. Bolded values with ** represent values that appeared significant. SE, standard error, CI 95, 95% confidence intervals, P Predictor, *p* value of clock used, EN Elastic Net, BLUP Best Linear Unbiased Prediction.


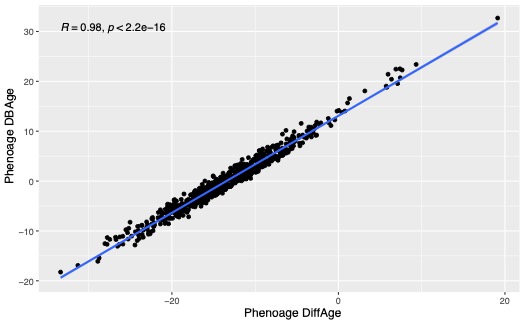


Supplementary Figure 3. Scatterplot of DiffAge (x axis) and DBAge (y axis) in AIBL calculated using the Phenoage Clock.

*R* = 0.98, *p* < 2.2e-16.


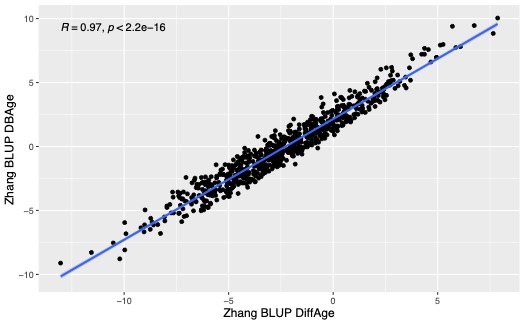


Supplementary Figure 4. Scatterplot of DiffAge (x axis) and DBAge (y axis) in AIBL calculated using the Zhang BLUP Clock. *R* = 0.97, *p* < 2.2e-16.


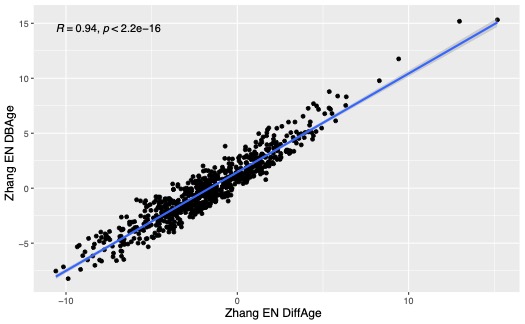


Supplementary Figure 5. Scatterplot of DiffAge (x axis) and DBAge (y axis) in AIBL calculated using the Zhang EN Clock.

*R* = 0.94, *p* < 2.2e-16.


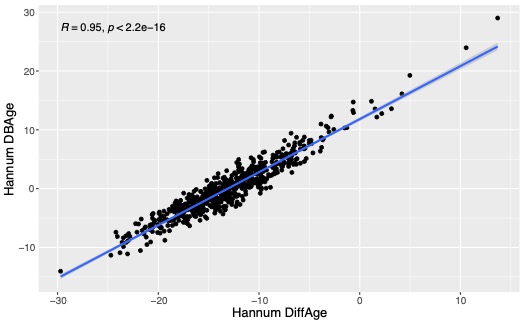


Supplementary Figure 6. Scatterplot of DiffAge (x axis) and DBAge (y axis) in ADNI calculated using the Hannum Clock.

*R* = 0.95, *p* < 2.2e-16.


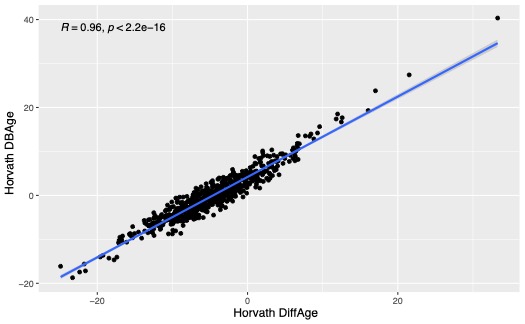


Supplementary Figure 7. Scatterplot of DiffAge (x axis) and DBAge (y axis) in ADNI calculated using the Horvath Clock.

*R* = 0.96, *p* < 2.2e-16.


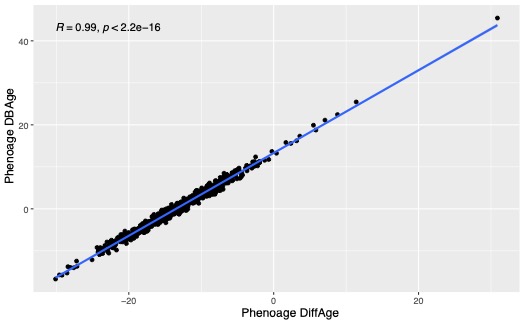


Supplementary Figure 8. Scatterplot of DiffAge (x axis) and DBAge (y axis) in ADNI calculated using the Phenoage Clock.

*R* = 0.99, *p* < 2.2e-16.


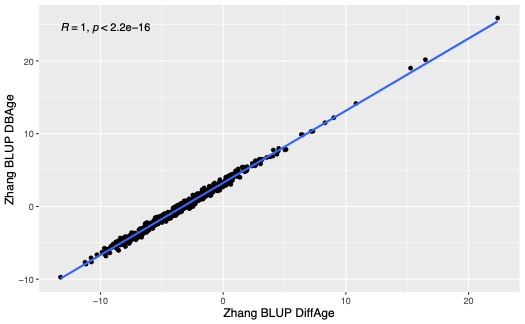


Supplementary Figure 9. Scatterplot of DiffAge (x axis) and DBAge (y axis) in ADNI calculated using the Zhang BLUP Clock. *R* = 1, *p* < 2.2e-16.


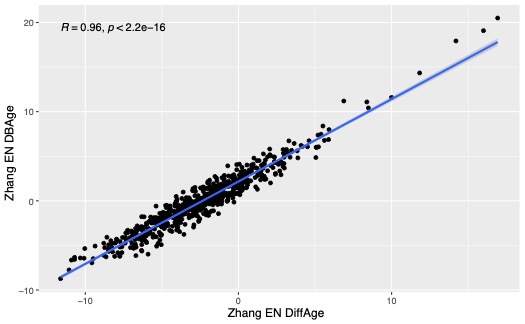


Supplementary Figure 10. Scatterplot of DiffAge (x axis) and DBAge (y axis) in ADNI calculated using the Zhang EN Clock. *R* = 0.96, *p* < 2.2e-16.
